# From Brewing to Plastic Degradation: Uncovering the Polyurethanase Potential of *R. chinensis* Lipase through Atomistic Simulations

**DOI:** 10.1021/jacs.5c21421

**Published:** 2026-02-19

**Authors:** Victor de Sousa Batista, Katarzyna Ṡwiderek, Vicent Moliner

**Affiliations:** BioComp Group, Institute of Advanced Materials (INAM), 16748 Universitat Jaume I, 12071 Castellón, Spain

## Abstract

In this computational study, we investigate the potential activity of a lipase produced by *Rhizopus chinensis* (RCL), a fungus traditionally used in Chinese brewing, to develop enzymatic biodegradation solutions of polyurethane (PUR), one of the most versatile and widely used synthetic polymers. MD simulations in water with low substrate concentrations confirm how the RCL adopts a closed conformation that restricts access to the active site. Upon substrate accumulation or an increase in substrate concentration , activation of the enzyme is achieved due to conformational changes involving not only the previously proposed rotation of Phe113 but also a significant rearrangement of Arg114. This movement induces the outward shift of Phe113, opening a channel for substrate entry. These findings support the classical interfacial activation mechanism of lipases and justify the selection of a quasi-open lid RCL model for modeling its catalytic activity. Using M06-2X:AM1/MM MD simulations, we validated the esterase activity of RCL on a benchmark ester compound 4-nitrobenzyl butyrate (pNPB), uncovering a standard acylation-hydrolysis mechanism with energy barriers (18.6 and 19.3 kcal/mol, respectively) in excellent agreement with experimental data. The urethanase activity of RCL was then explored in the degradation of a model substrate, 4-nitrophenyl benzylcarbamate (pNC). Our results indicate that the process preferentially follows an esterase pathway, fully decomposing the PUR-like model sample through three steps: acylation, hydrolysis, and decarboxylation. The value of the activation energy of the full process, determined by the acylation step (17.2 kcal/mol), indicates a feasible reaction. Comparative analysis between the degradation of pNPB and pNC reveals that RCL’s catalytic efficiency is influenced by the geometry and electrostatic nature of the substrate, with the enzyme’s active site aligning key moieties for effective bond cleavage. Additionally, short-range interactions, along with long-range electrostatic effects, polarize key moieties, facilitating charge redistribution during bond formation and cleavage. Our findings provide valuable insights into RCL’s potential as a biocatalyst for PUR degradation and suggest that redesigning the enzyme may include not only mutations to decrease the activation energy of the chemical steps but also increasing the population of polymer-accessible protein conformations.

## Graphical abstract

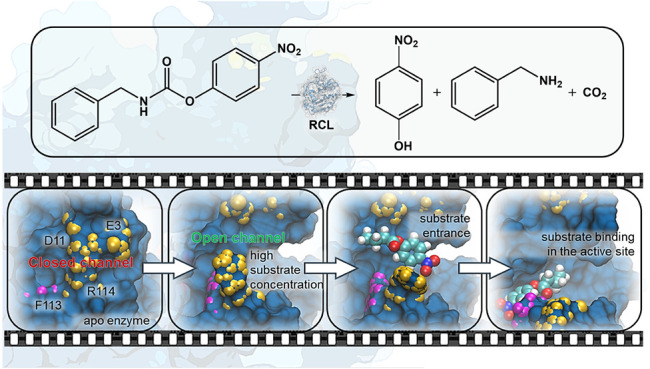

## Introduction

Plastic pollution is a global environmental issue that arises from the widespread use and improper disposal of exploited plastic materials, posing a significant threat to biomes, marine life, and human health. Plastic materials are synthetic polymers that have a persistent nature, lingering in the environment for extended periods and causing potential long-lasting and irreversible impacts once they surpass critical thresholds.^1^ Furthermore, extensive utilization of plastics, associated with their slow degradation and poor waste mechanical management, results in the pervasion of microplastics in virtually all of Earth’s environments, with recent studies detecting their presence inside the human body.^2–5^ One way of dealing with plastic waste is to increase plastic circularity by recycling and upcycling. Plastic recycling involves collecting, sorting, cleaning, and transforming discarded plastic materials into their constituent monomers, which can be accomplished mainly through mechanical/thermal approaches or chemical processes, often combining them. Recycling helps to conserve natural resources and reduce the demand for virgin plastic production, but nowadays, only about 9% of the yearly global plastic emissions get recycled.^6,7^ Given the current expanding trend of plastic waste production, it becomes imperative to devise new recycling strategies and methods.

Enzymatic catalysis is gaining popularity as a recycling method.^8^ Through the depolymerization of plastics, it becomes feasible to either regenerate the same type of plastic (recycling) or convert it into other added-value chemicals (upcycling).^9^ Additionally, enzymatic depolymerization operates under significantly milder pressure and temperature conditions when compared to nonenzymatic chemical methods.^10^ The interest in the enzymatic degradation of synthetic polymers has grown progressively in recent years, with several polymer-degrading enzymes and organisms being discovered.^11–19^ However, this approach has no one-size-fits-all solution, as each plastic has its specific chemical characteristics, especially regarding its chemical bonds and the nature of the interactions between the biocatalyst and the polymer, and thus requires a case-by-case study. Most of the progress made toward the use of enzymes to degrade plastics targets polyesters, such as polyethylene terephthalate (PET) and polylactide (PLA), while searching for enzymes that degrade polyurethane (PUR), polyethylene (PE), polypropylene (PP), or polyvinyl chloride (PVC) remains a task of utmost importance.^20^ The current challenges associated with the enzymatic degradation of synthetic polymers still hamper the widespread use of the technique.^21^ Such challenges include the slow pace at which most of these reactions occur, the enzyme’s poor tolerability for harsher reaction conditions, the insolubility of plastics in water, and, finally, the limited scope of polymers that can currently be degraded by the known biocatalysts. Nevertheless, this innovative technique holds great promise in revolutionizing the recycling industry, as it offers a more sustainable and resource-efficient solution to the global plastic waste crisis, making research in the field highly valuable.^22^

Of all types of plastics, PURs are among the most versatile and widely used materials due to their durability, flexibility, and insulation properties. PURs are the sixth most common plastic, with consumption reaching more than 20 Mt per year.^23^ Indeed, after polyesters, PURs are the second largest class of hydrolyzable plastics produced, which makes them a target for recycling by chemical approaches.^10^ However, the chemical recycling of PURs, unlike PET recycling, is poorly developed, since PUR depolymerization processes require very drastic conditions, mainly due to its high chemical complexity (several types of bonds; ureas, urethanes, ethers, esters, allophanates, etc.). Hence, the resulting monomers after depolymerization processes are of low purity or incapable of reacting in a new polymerization cycle. Recycling PURs is also challenging due to their thermosetting nature, which makes them resistant to melting and solvent dissolution. Mechanical recycling of PURs involves grinding and shredding waste plastic into smaller particles, which can then be used as fillers or additives in new materials, but this results in downcycled, less valuable products. Chemically recycling PURs through depolymerization reactions such as hydrolysis, aminolysis, acidolysis, and glycolysis has the potential to produce plastics with equal or higher aggregate value, but these processes are energy-intensive, often requiring high temperatures and pressure.^24^

Recently, the degradation of PURs by biocatalytic approaches has been gaining more attention,^25^ mostly through the identification and characterization of microorganisms and pure enzymes capable of cleaving key bonds in PURs.^8^ Several microorganisms, mainly fungal and bacterial strains, have been reported to degrade low molecular weight urethane-based model compounds, although the enzymes responsible for such hydrolytic activity were, in many cases, not fully characterized or even identified. On the other side, several types of enzymes, such as promiscuous esterases, lipases, cutinases, proteases, amidases, ureases, and oxidases, have been shown to cause some degree of degradation on PURs, but the processes are extremely slow and achieve only minimal degradation.^8,22^ The enzymes capable of breaking down PURs are frequently referred to as polyurethaneases (PURases), although this label may be misleading as it focuses on their activity on PURs rather than on their specific capacity to hydrolyze urethane bonds.^8^ Indeed, the PURases reported so far refer to hydrolases, like lipases or cutinases, that act mostly on the ester bonds in polyester-PURs.^26–29^ Examples of PURases capable of cleaving urethane bonds in polyether-polyurethanes have remained inaccessible to biocatalytic hydrolysis, having only been recently described, and they are only effective after glycolysis of nonurethane bonds and on small molecular weight carbamates.^30,31^ Recently, a variety of hydrolases (lipases, proteases, cutinases, urethanases) were screened for their ability to catalyze the transcarbamoylation of PUR thermosets in primary alcohols under mild conditions using methanol and ethanol as reaction agents, representing an innovative strategy.^32^

Given the rising demand for PURs, the global concerns over plastic waste, the challenges in recycling these materials, and the potential advantages of enzymatic catalysis in PUR recycling, it is evident that the search for novel, true PURases is of paramount importance.^33^ Currently, many PUR-degrading lipases have been identified from *Pseudomonas fluorescens* (PuIA),^34^ *Pseudomonas chlororaphis* (pueA and pueB),^35^ *Candida rugosa*,^36^ *Pseudomonas protegens* strain Pf-5 (pueA and pueB),^37^ most of these studies using Impranil, a commercial PUR sample, to verify enzymatic PURs degradation. Furthermore, Bayer has filed two patents for the use of enzymatic processes to degrade a series of plastics, including PURs, which include *Candida antarctica* lipase B (CALB) and *Aspergillus niger* lipase.^38,39^ However, despite being discovered long ago, these enzymes still require further detailed characterization to quantify and compare their activity on different types of PURs and to improve their proficiency for future industrial applications. More recently, a unique and efficient PURase, PufH, from a novel *Pueribacillus* sp. YX66 was identified.^40^ The enzyme was shown to degrade commercial polyurethane foam (PUF). However, its mechanism of action is consistent with the cleavage of the plastic’s ester bond, showcasing how elusive selectively cleaving the urethane bond can be.

We studied the depolymerization of PET by hydrolases from *Ideonella sakaiensis* 201-F6 and the ICCG variant of the metagenome-derived leaf-branch compost cutinase (LCC),^41,42^ and more recently by CALB.^43^ In this last study, we exploited the results of the pH effect to propose a pH-controlled biotransformation that selectively hydrolyzes bis­(hydroxyethyl) terephthalate (BHET) to either its corresponding diacid or monoesters using both soluble and immobilized CALB.^43^ These studies have been extended to the depolymerization of Impranil, as a model of PUR samples, by PueA, which allowed confirming the polyurethane esterase activity of wild-type PueA, although at low-level.^44^ Recently, insights into the activity of a metagenome-derived urethanase discovered by Bornscheuer and coworkers,^30^ UMG-SP2, catalyzing the degradation of a urethane-like model compound, 4-nitrophenyl benzylcarbamate (pNC), revealed an esterase-like three-step mechanism, acylation, hydrolysis, and decarboxylation, with this particular substrate.^45^ Interestingly, despite acylation and hydrolysis appearing to be kinetically and thermodynamically feasible, the decarboxylation showed a low energy barrier but with an endergonic character.

Following these studies, here we use molecular dynamics (MD) simulations with molecular mechanics (MM) and quantum mechanics/molecular mechanics (QM/MM) potentials to study the viability of a *Rhizopus chinensis* lipase (RCL), a fungus traditionally used in Chinese breweries, as a candidate to serve as PURase. This enzyme has been successfully employed to perform esterification reactions on short-chain fatty acids in industrial settings, both in the presence and absence of organic solvents.^46^ Its widespread use and accessibility, together with its favorable chemical properties, make it a promising candidate for preliminary biodegradation studies using PUR oligomers. Thus, the first step of our study was the validation of our methodology by studying the esterase activity of RCL with the benchmark ester compound 4-nitrobenzyl butyrate (pNPB), commonly used to study lipase activity, including RCL.^47^ Subsequently, a model PUR dimer compound, 4-nitrophenyl benzylcarbamate (pNC), was used as a substrate to investigate the potential PURase activity of RCL, both as an esterase and amidase (see Figure 1). Despite the lack of an aliphatic alcohol moiety, frequently present in PUR-based plastics,^48,49^ the selection of pNC as a PUR model compound was motivated by computational and experimental considerations. First, the use of a small model allows including the full molecule in the quantum region during QM/MM simulations, thereby avoiding a possible source of errors derived from, for instance, introducing additional quantum link atoms. Beyond this computational advantage, additional arguments support the suitability of pNC as a representative model of real PUR systems. Thus, pNC is a small urethane surrogate that resembles the aromatic amines found in polyurethanes. Indeed, pNC has been employed in our recent studies on the exploration of urethanase activity of UMG-SP2 in water and on the screening for activity of variety of hydrolases (lipases, proteases, cutinases, urethanases) to catalyze the transcarbamoylation of PUR thermosets in primary alcohols under mild conditions.^32^ Finally, as shown in Figure 1, the model compound contains an asymmetric carbamate bond that, upon hydrolysis, releases two products readily detected by UPLC-MS methods. This feature facilitates experimental validation and is of particular value for the scientific community interested in benchmarking and following up on our computational predictions. Overall, the results obtained from the simulations and subsequent bioinformatic analyses can provide a solid foundation for future protein engineering efforts aimed at enhancing the polyurethanase activity of RCL.

**Figure 1:**
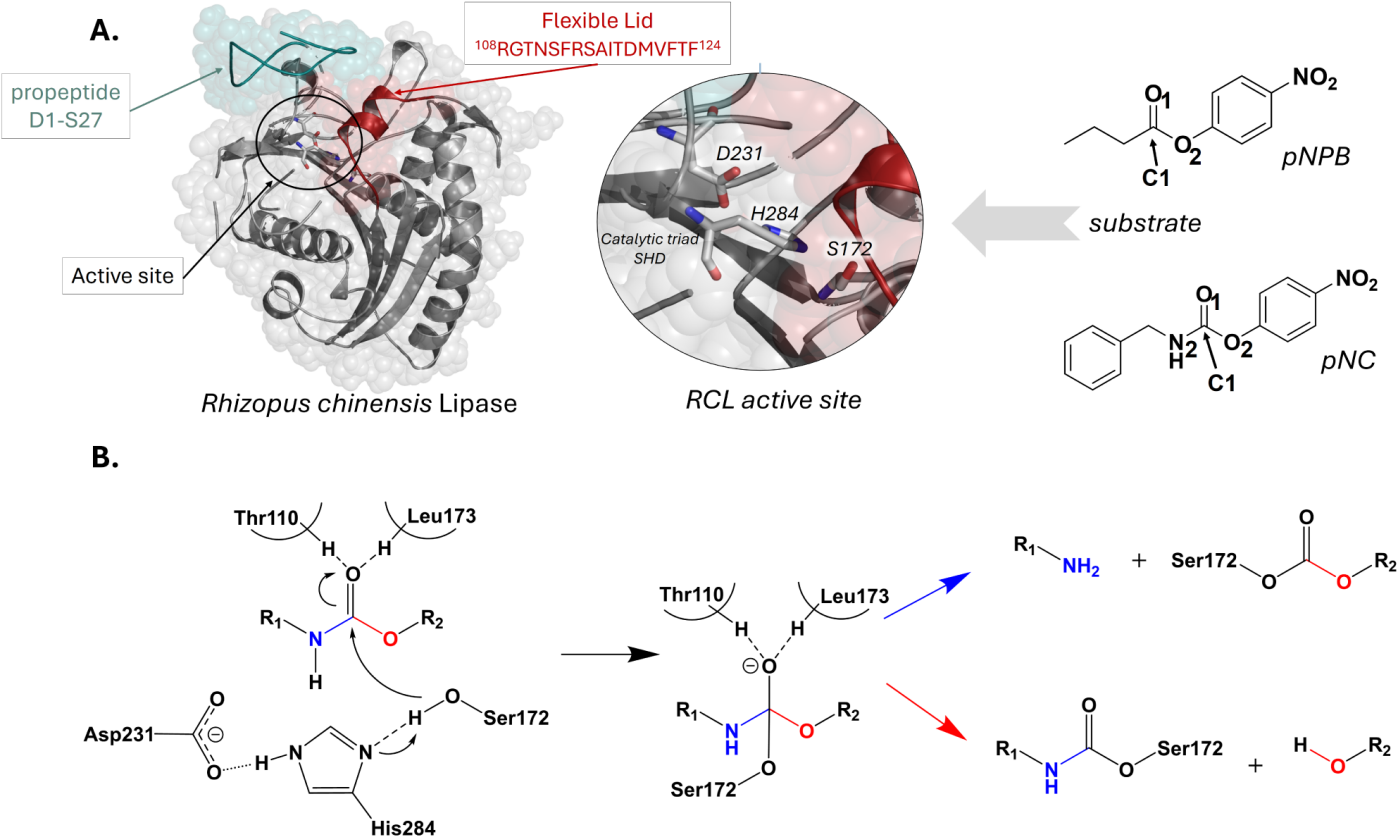
A. Complete structure of the RCL (PDB 6A0W) with incorporated missing protein fragments, the active site structure with highlighted catalytic triad residues, and model compounds employed as substrates to study the reactivity. B. Schematic representation of the acylation step of the possible amidase (blue arrow) or esterase (red arrow) activity of RCL.

## Methodology

### System Setup and MD Simulations

The molecular model used in the computational studies was derived from the crystal structure of the apo form of the RCL (PDB ID: 6A0W), which exhibits the enzyme in its closed-lid conformation.^50^ The missing protein fragments, such as amino acid residues 1, 24–31, and 297–305, were added using Discovery Studio Visualizer (DSV).^51^ Before proceeding with further preparation protocol, 500 ps of gas-phase MD simulations were performed using NAMD (ver. 3.0)^52^ with only the atoms from the added residues allowed to move. Protein parameters were obtained from the AMBER ff03 force field (FF).^53,54^

As shown in Figure 1A, although the catalytic triad residues Ser172, Asp231, and His284 are located at their canonical positions in the α/β hydrolase fold, the lid region, i.e., residues Gly109–Thr123, with a short α-helix formed between Phe113 and Asp119 linked to the “core” of the protein structure, completely covers the active site pocket, preventing substrate docking. The relevance of the movements of this lid in RCL’s interfacial activation and thermostability has been previously demonstrated by kinetics and protein disulfide engineering experiments.^50,55^ Thus, to construct an open conformation variant enabling substrate binding, we searched for proteins exhibiting a similar three-dimensional structure to RCL with the lid in an alternative orientation. Using VAST+^56^ software, the existence of several proteins that share a similar secondary structure to RCL was revealed. Six structures of different origins with significantly high RMSD (from 1.90 to 2.55 Å) values and a high number of aligned residues (from 236 to 255), as listed in Table S1, were selected for further examination. All identified proteins exhibit low amino acid sequence identity with RCL. However, despite the highest sequence identity being only 32%, the overall backbone arrangement, excluding the lid structure, is nearly identical in three-dimensional space. Three structures of lipase from *Gibberella zeae*, *Yarrowia lipolytica*, and *Penicillium cyclopium* (PDB ID 3NGM,^57^ 3O0D,^58^ 5CH8,^59^ respectively) provided a perfect overlay with RCL, while the remaining three, lipase from *Malassezia globosa*, *Thermomyces lanuginosus*, and *Aspergillus niger* (PDB ID 5GW8,^60^ 1DTE,^61^ 1USW,^62^ respectively) exhibited open-lid conformation, as shown in Figure S1. Because fungal lipase from *Thermomyces lanuginosus* (TLL) crystallized both in open (PDB ID: 1DTE) and closed-lid (PDB ID: 1DT5 ^62^) conformations (see Figure S2), with the closed variant perfectly overlaying with RCL structure, we assumed that RCL would behave similarly under different conditions, as explained by Patkar and coworkers.^61^ Using the open TLL variant as a reference, it was possible to build an open lid conformation of RCL by superimposing both proteins, then substituting residues 108–124 (^108^RGTNSFRSAITDMVFTF^124^) of RCL with residues 81–97 (^81^RGSRSIENWIGNLNFDL^97^) of TLL, corresponding to the lid amino acids, and finally mutating these residues back to those of RCL. This new RCL conformer was then used to dock the reference ester pNPB using AutoDock Vina,^63,64^ taking the Cartesian coordinates of protein atom OG_Ser172_ as a center of a 10 × 10 × 10 Å^3^ search box. In AutoDock Vina, partial atomic charges of amino acids and pNPB were assigned explicitly to each atom based on the Gasteiger model.^65^

Open-lid RCL model with bound pNPB substrate (Figure S3 in the Supporting Information) was subsequently used in a Generalized Born implicit solvent (GBIS)^66^ MD simulation using the NAMD (ver. 2.0)^67^ engine to examine the behavior of the open lid upon solvent effect. To set up the system, the p*K*_*a*_ values of the titratable residues were determined using the empirical program PropKa v.3.0.3.^68^ The results are provided in Table S2 of the Supporting Information. The protonation state of each titratable residue was assigned according to the selected experimental conditions of pH 8.5. Residues Glu292 (p*K*_a_ of 9.86), His300 (p*K*_a_ of 8.61), and His302 (p*K*_a_ of 8.66) were predicted to be in nonstandard protonation states and thus were protonated accordingly. Additionally, a visual inspection was performed to identify potential hydrogen bond contacts established between the titratable residues and neighboring amino acids in the protein. Due to the adopted intramolecular interactions network, histidines His136, His171, His224, His245, His301, His303, His304, and His305 were protonated in δ, while His228, His235, and His284 in ε positions. Additionally, three disulfide bridges between Cys56 and Cys295, Cys67 and Cys70, as well as Cys262 and Cys271, were identified and treated accordingly with the selected force field (FF). Missing parameters for pNPB (see Table S3 in Supporting Information) were generated with Generalized Amber Force Field (GAFF2)^69^ using the Antechamber^70^ software, with atomic charges computed employing the AM1-BCC^71^ method according to the standard procedure used in our previous related studies.^72–74^ Hydrogen atoms were added using AmberTools LEAP module. Protein parameters were the same as described above for the gas-phase MD. The prepared model underwent energy minimization using a conjugate gradient algorithm, followed by heating to 313 K in 0.1 K increments and a 300 ns NVT MD simulation. During these simulations, the distance between the oxygen OG from Ser172 and the carbonyl carbon C1 from the substrate was constrained at 3.2 Å with 150 kJ/mol·Å^2^ force constant to avoid its possible diffusion from the open active site. Additionally, the coordinates of the pro-peptide region were kept frozen to avoid perturbation originating from its possible interactions with the lid during simulation, as described elsewhere.^47^ GBIS MD simulations were performed in two solvent models, water (with a dielectric constant (ε) of 78.5 and ionic strength of 0.2) and hexane (ionic strength of 0.0 and ε of 1.88).

The final structure obtained after the simulation in water was used as a starting point for longer unconstrained MD simulations of pNPB-RCL using explicit solvation, as well as the initial geometry to bind the pNC substrate. pNC compound was built manually using the equilibrated position of the pNPB substrate in the active site as a pattern. As explained in the next section, two different poses of the substrate in the enzyme active site, pNC-RCL_A_ and pNC-RCL_B_, were selected. Because of the relative orientation of the functional group with respect to the active site catalytic residues, the two poses could render two alternative esterase and amidase activities. Missing parameters for the pNC (see Table S4 in Supporting Information) were generated according to the same procedure as described for pNPB. Finally, the three generated models were subsequently neutralized by adding one chloride ion at an electrostatically most favorable position and solvated in a water box with 10 Å set as the buffering distance between the edges of the box and the protein. The parameters for chloride ions and water molecules were adapted from the TIP3P^75^ FF. After optimization, systems were gradually heated to 313 K in 0.1 K increments and underwent 500 ps of NPT equilibration. The last snapshot obtained after the equilibration step was then used as an initial geometry for three independent MD simulations, with the temperature being controlled using the Langevin thermostat^76^ and the pressure with the Nosé–Hoover Langevin piston.^77^ Periodic boundary conditions (PBC) were used, and the nonbonded interactions were treated with a smooth switching function applied between 14.5 and 16 Å using Particle-Mesh Ewald (PME) summation. In all classical MD simulations done using NAMD (ver. 3.0), a 2 fs time step was used through the SHAKE algorithm.^78^ Unconstrained NPT MD simulations of 500 ns were done with 3 independent replicas being evaluated for each system. Time evolution of the root-mean-square deviation (RMSD) computed for the backbone atoms (C, O, Cα, and N) of protein and substrate heavy atoms was analyzed to confirm the stability of the reactant-complex structures (Figure S4 in Supporting Information). The time-dependent evolution and the distribution of the distances between the atoms involved in the reaction mechanisms, as well as the Bürgi–Dunitz angle (α_BD_), were also evaluated (Figures S5–S8 in the Supporting Information). In addition, the population and time-dependent behavior of the Phe113 side-chain conformers were evaluated under different conditions (Figures S9–S10). Moreover, the interaction energy between the protein and substrate was calculated (Figure S11–S13). All data processing and analysis were done using cpptraj package.^79^

### QM/MM Potential Energy Surfaces (PES)

To study the efficiency of RCL in hydrolyzing both selected substrates, the most populated reactive conformations of the pNPB-RCL, pNC-RCL_A_, and pNC-RCL_B_ systems were selected based on the distribution of distances between the atoms involved in the reaction mechanism, i.e., the reaction coordinates representing the higher populated configurations during MD simulations. As shown in Figure 1, it was initially assumed that the reaction with the benchmark ester pNPB proceeds by an initial acylation step followed by a deacylation or hydrolysis step, as observed for other serine hydrolases.^80,81^ In the case of the polyurethane-like model, pNC substrate, both esterase and amidase activities were explored, depending on the substrate bond breaking (C–O or C–N) that occurs after the nucleophilic attack of Ser172 to the carbonyl carbon atom of the substrate.

In this study, an additive hybrid QM/MM approach with an electrostatic embedding was used to construct the total Hamiltonian, employing quantum link atoms for the QM-MM frontier treatment.^82^ The QM subset of atoms (see Supporting Information for details) was treated initially with the AM1 ^83^ semiempirical Hamiltonian (low level, LL) and later corrected with the M06-2X^84^ functional and the 6-31+G­(d,p) basis set (high level, HL) as implemented in Gaussian 09.^85^ The remaining protein and solvent molecules were treated using AMBER and TIP3P classic force fields, respectively. The positions of the atoms beyond 25 Å from the substrate were fixed, and the nonbonding interactions were treated using the same cutoffs as in the classical MD simulations. All QM/MM calculations were performed using the fDynamo library.^86,87^ Before each step, the structures selected as starting points were optimized at the LL/MM level using a combination of the conjugate gradient and L-BFGS-B^88^ algorithms with gradient tolerance set to 0.1 kJ·mol^–1^·Å^–1^. Then, QM/MM Potential Energy Surfaces (PES) were explored by choosing and scanning the appropriate combination of internal coordinates (ζ_*i*_), assuming their relevant role in the shape of the reaction coordinate (see Supporting Information for details). Transition state (TS) structures were localized with a micromacro iteration optimization algorithm^89,90^ together with Baker’s algorithm^91,92^ at M06-2X/MM. The nature of the TSs was confirmed by frequency calculation, observing only one imaginary frequency value (see Tables S14–S20 of the Supporting Information). Then, the Intrinsic Reaction Coordinate (IRC)^93,94^ paths were traced down from the located TSs to the connecting valleys in mass-weighted Cartesian coordinates. The same optimization strategy and frequency analysis were employed to characterize each minimum, ensuring that no imaginary frequencies were found.

### QM/MM Free Energy Surfaces

Free Energy Surfaces (FESs) were obtained in terms of one- or two-dimensional potential of mean force (1D- or 2D-PMF)^95^ for the acylation, hydrolysis, and decarboxylation steps. In all cases, Umbrella Sampling (US)^96^ was used in combination with the Weighted Histogram Analysis Method (WHAM),^97^ where the grid points for each FES came from its respective PES. A series of QM/MM MD simulations was performed at LL/MM, adding an umbrella force constant constraint of 2500 kJ·mol^–1^·Å^–2^ for the selected reaction coordinates. The simulations were performed with a total of 5 ps of equilibration and 20 ps of production at 313 K for pNPB-RCL, pNC-RCL_A_, and pNC-RCL_B_ models. Window overlapping analysis was carried out to confirm the convergence of the resulting free energy surfaces (Figures S18–S20 of Supporting Information). The temperature was controlled with the Langevin–Verlet algorithm,^98^ and the time step was 1 fs. To improve the quality of the results obtained with the LL simulations, corrections were made at HL by means of spline interpolation. In such corrections, the final energy was obtained from a correction term computed using the single-point energy difference between the HL and LL for the QM subset of atoms.^99,100^

## Results and Discussion

### Open-Lid vs Closed-Lid Conformation

As mentioned in the Introduction section, the main goal of this research was to showcase the potential of the RCL enzyme as a PURase by providing an atomistic insight into its cleaving capacity toward a small PUR model compound (pNC). Before, however, its esterase activity was tested with a similar compound, pNPB, for which experimental data are available.^47^ To achieve these objectives, it was essential to generate a reasonable initial pose of the substrate within the binding pocket, which could be accomplished using molecular docking tools followed by MD simulations. Unfortunately, the RCL crystal structure retrieved from the crystallographic experiments shows a closed-lid conformation where the catalytic triad residues Ser172, His284, and Asp231 are buried inside the protein, inaccessible to the solvent and thus unable to bind any substrate and catalyze its hydrolysis.^50,101^ Thus, as explained in the Methodology section, an open-lid variant of RCL was built and used to dock pNPB, followed by 300 ns of GBIS MD simulations in water. The binding pose of pNPB was selected for the MD simulations based on the structure with a close distance observed between the atoms involved in the acylation reaction steps, i.e., OG_Ser172_–C1_pNPB_ and NE2_His284_–O2_pNPB_ (respectively 4.04 and 4.66 Å), see Figure S3 in Supporting Information.

As expected, the lid domain underwent a significant conformational change during MD simulations in aqueous media, transitioning from the initial open to the final closed state, as illustrated in Figure 2A. This was evidenced and quantified by the significant changes in the RMSD values computed for the backbone atoms of this highly flexible fragment (magenta line in Figure 2B). Complementarily, GBIS MD simulations using hexane as solvent confirmed that the lid domain does not close in a nonpolar medium, as proved by the small oscillation of RMSD values (gray line in Figure 2B). The substrate was sandwiched between the lid and the bulk of the protein in closed-lid conformation, as shown in Figure 2C. This dramatic change and reduction of the binding cavity size suggest that the lipase is inactive in polar conditions. However, upon overlaying the simulated lid obtained in GBIS MD in water onto the initial RCL crystal structure, it was observed that while the lid adopts a close conformation in both structures, Phe113 shows an alternative, outward-facing orientation in our simulations, as illustrated in Figure 2D. The outward orientation, characterized by dihedral φ_Phe113_(C–C_α_–C_β_–C_γ_) of ca. −60°, ensures open access to the active site by removing the steric hindrance caused by the bulky aromatic side chain and provides a solvent-accessible active site, even in a closed-lid conformation (φ_Phe113_ of ∼180°). This “quasi-open” conformation of the active site is sufficient to allow substrate access to the catalytic residues. However, it cannot be excluded that the new Phe113 position observed in simulations is due to the presence of the substrate initially posed in the active site. Thus, to dispel all doubts related to the Phe113 orientation in RCL during substrate binding, additional simulations for the apoenzyme were performed to understand the mechanism of Michaelis complex formation.

**Figure 2:**
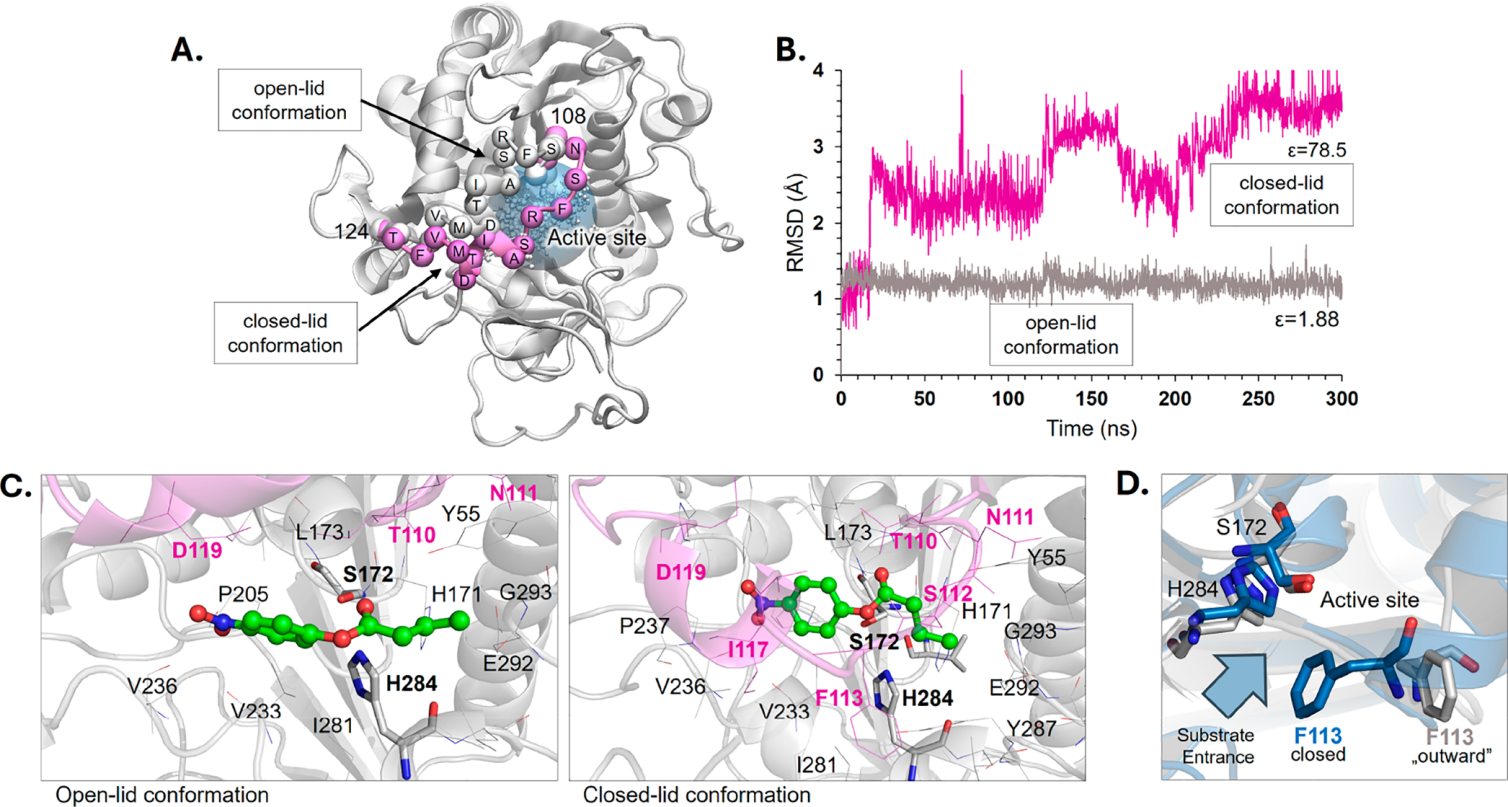
Solvent effects on the structure of RCL. A Overlay of the lid secondary structure in aqueous solvent before (in silver) and after 300 ns of GBIS MD simulation (in magenta). B. Time evolution of RMSD computed for backbone atoms of the lid (residues 108–124) along the MD simulation in water (in magenta) and hexane (in gray) solvent. C. Structural rearrangement of the active site and pNPB substrate (in green) upon water solvent effect. The lid structure is highlighted in magenta. D. Alternative closed and “outward” Phe113 orientations in the RCL crystal structure (PDB ID 6A0W) (in blue) and RCL variant obtained after 300 ns of GBIS MD simulation in water (in gray).

### Quasi-Open-Lid Stabilization and Accessibility of the Substrate to the Enzyme Active Site

Comparative studies were conducted using two models: the RCL apoenzyme without substrate molecules in the environment, and the apoenzyme with 40 pNPB molecules located near the Phe113 residue, used to mimic the specific composition of the microenvironment with the critical micelle concentration of the substrate. Both models were used to evaluate whether high concentrations of pNPB near the binding cavity could induce and stabilize conformations of Phe113 that promote channel opening, even under polar solvent conditions. In fact, as previously proposed by Yu and coworkers, the interfacial activation mechanism must be related to conformational changes of the Phe113 side chain in the presence of high amounts of substrate.^102^ The rotation of this residue is proposed to create a cavity large enough to accommodate substrate binding. However, the complete mechanism linking this rotational movement to substrate binding remains unknown.

To shed light on this process, the representative conformation of the protein, characterized by the side chain of Phe113 adopting an outward configuration (φ_Phe113_ ∼ −60°), was extracted from the structures previously generated during GBIS MD simulations of the RCL apoenzyme in water. This conformation was selected due to its potential relevance for substrate access to the active site, and it was used in MD simulations with an explicit water model, with and without the addition of pNPB molecules. pNPBs were spatially arranged around the protein surface using the Packmol software,^103^ which enables the construction of initial molecular configurations by placing molecules into defined regions. Following this automated packing step, the positions of the pNPB molecules were refined manually using Discovery Studio Visualizer. Manual adjustment was performed to ensure an increased concentration of substrate molecules in the vicinity of functionally important regions of the enzyme, namely, the lid and propeptide domains. These domains are believed to play a role in substrate recognition and gating,^50^ and concentrating pNPB molecules in their proximity allowed for a more targeted sampling of potential binding events during subsequent simulations.

Based on the results of MD simulations obtained from three independent 500 ns replicas performed in the absence of substrate in the solvent, it was confirmed that the active site remains closed in the apoenzyme, with a Phe113 conformer characterized by φ_Phe113_ oscillating around 180°. 89% of the structures were found in this conformation, as shown in Figure 3A. Time dependent population analysis, provided in Figure S10A of the Supporting Information, confirms the reliability of these data. This protein model without substrate molecules experienced no significant changes in the RCL structure throughout the 500 ns trajectories. As expected, immediately upon initiation of the simulations, the gate to the active site was occupied by the Phe113 aromatic ring, blocking possible direct access.

**Figure 3:**
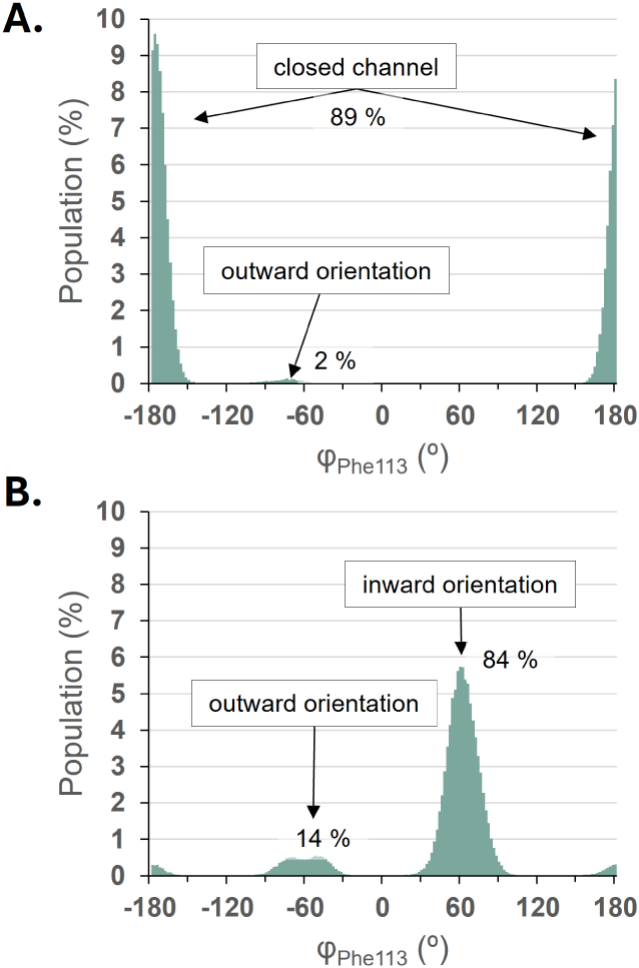
Population analysis of Phe113 conformers identified based on the φ-dihedral angle (C–Cα–Cβ–Cγ) in the 150,000 structures generated during 3 replicas of 500 ns MD simulations of RCL without A. and with B. addition of a high concentration of pNPB substrate.

This scenario completely changed at a high concentration of substrate, when pNPBs approach the lid and propeptide regions. In this case, the equilibrium was shifted toward a quasi-open active center, which now exhibits distinct inward Phe113 orientations, predominating in 84% of all observed instances (see Figure 3B and Figure S9A for details). Thus, in the presence of the micelle concentration of substrate, Phe113 adopted an orientation that is different from the initial position, now facing inside the active site with a conformation characterized by φ_Phe113_ of +60°, but equally exposing an open channel for substrate access to the active site. This reorientation can be attributed to the loss of the strong hydrogen bond interactions initially formed between Arg114 of the lid and Glu3 and Asp11 of the propeptide, which are maintained in the unperturbed RCL model.

Overall, the results analysis of these MD simulations allowed us to identify four consecutive steps required for opening of the active site and binding the substrate, as illustrated in Figure 4A. Evolution of key parameters such as distances between carbon CZ of Arg114 and CD of Glu3 or CG of Asp11, dihedral angle (φ_Phe113_), and distance between carbonyl carbon C1 of pNPB and OG of Ser172, as shown in Figure 4B, confirms existence of all described states. An animation depicting the entire binding process has been provided in the Supporting Information Movie S1 to enhance the reader’s understanding.

**Figure 4:**
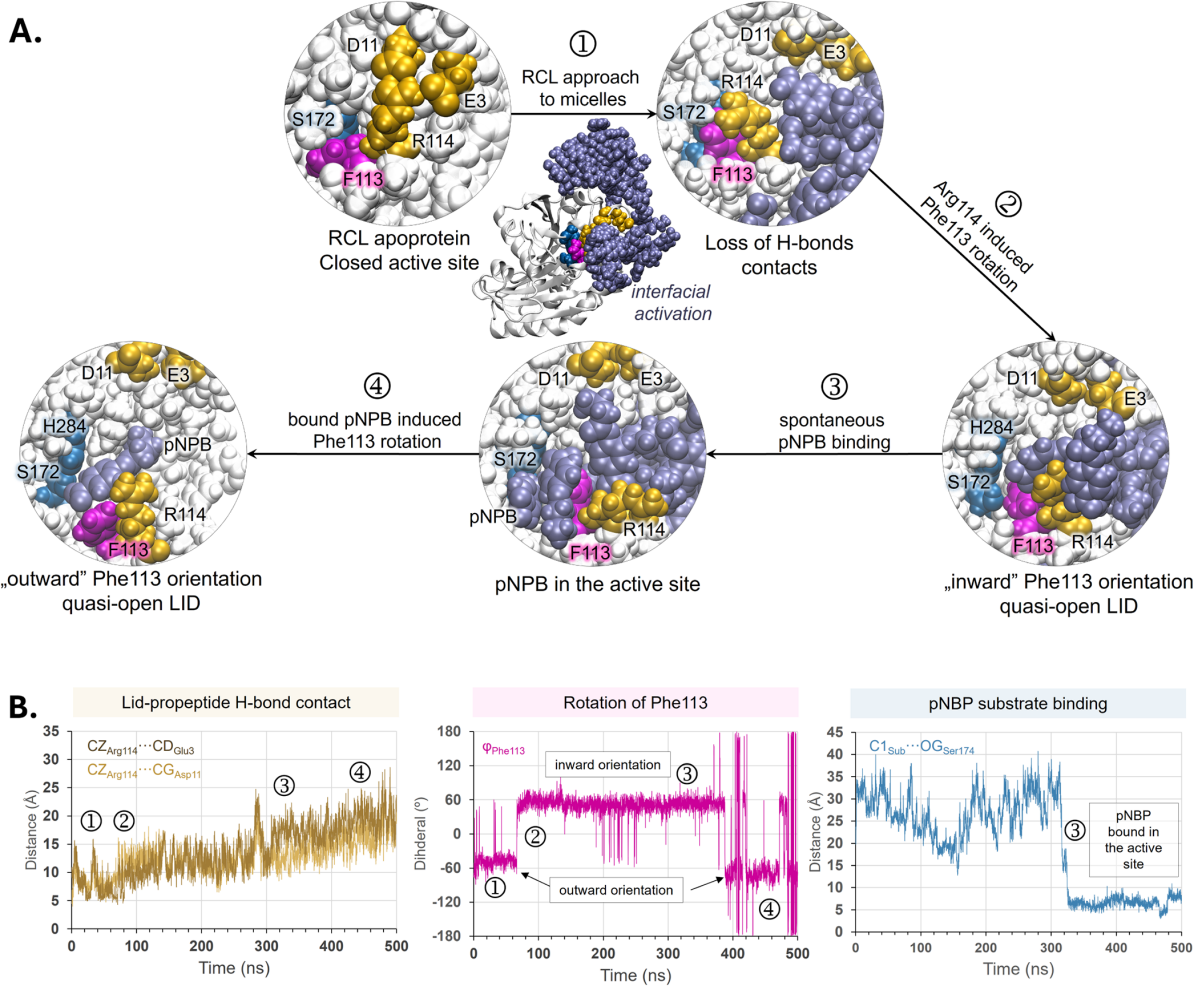
A. Four steps of the spontaneous substrate binding mechanism deduced from 500 ns of unbiased MD simulations of RCL in a simulated micellar concentration of pNPBs: ① Approach of the apoprotein to the pNPB aggregate disrupts the original hydrogen bonds between Arg114 of the lid and Glu3/Asp11 of the propeptide; ② Reorientation of Arg114 induces an “inward” orientation of Phe113; ③ The “inward” conformation of Phe113 facilitates pNPB binding in the active site; ④ Binding of pNPB in the active site triggers reorientation of Phe113 to the “outward” conformation. B. Evolution of key parameters: distance between CZ of Arg114 and CD of Glu3 and CG of Asp11 (left panel), dihedral angle, φ_Phe113_ (center panel), and distance between C1 of pNPB and OG of Ser172 (right panel). The appearance of each stage throughout the MD simulations is highlighted.

As revealed by the analysis of the system’s time evolution, activation of RCL requires conformational changes involving not only the previously proposed movement of Phe113 but also a significant rearrangement of Arg114. The reorientation of Arg114 appears to be driven by the entry of pNPB molecules into the cavity formed between the propeptide and lid regions. The presence of pNPB molecules disrupts the strong hydrogen bonds and Coulombic interactions initially established between the positively charged Arg residue and the negatively charged Asp11 and Glu3 residues. The induced rotation of the highly hydrophilic arginine side chain toward Phe113 compels the latter to shift toward a more hydrophobic region, i.e., the interior of the active site. The resulting displacement of the bulky group exposes the catalytic residues, facilitating their engagement with the substrate. This is confirmed after 300 ns of classical MD simulations, ③ in Figure 4, when one of the substrate molecules binds within the active site near the catalytically relevant Ser172, remaining bound throughout the final 100 ns of the simulation. In the presence of pNPB within the binding pocket, Phe113 adopts an outward orientation, confirming that the position of this phenyl ring is sensitive to active site occupancy, which is consistent with the results of GBIS MD simulations done for the RCL-pNPB complex in an implicit water model. The fact that the described conformational changes take place spontaneously and consistently in our unconstrained MD simulations carried out at 313 K, suggests exergonic processes without significant energy barriers.

To support the proposed role of Arg114, three independent replicas of 500 ns MD simulations were performed at high substrate concentration, in which the initial hydrogen-bond interactions with Asp11 were restrained. The results reveal how the lid is not opened in a nonpolar environment if these interactions are preserved (see Figure S10B in Supporting Information).

The different behavior of the lid observed in polar and nonpolar conditions agrees with the classical interfacial activation mechanism of lipases.^101^ Therefore, based on the obtained results, it can be concluded that in aqueous conditions with low concentration of substrate molecules, the RCL closed conformation of the lid dominates. As the concentration of substrate increases, which can be considered equivalent to the formation of micelles, an interface is created that stabilizes substrate accessible active site conformations by rotation of Phe113, as suggested based on experimental evidence by Montelione and coworkers.^50^ Interestingly, by measuring the initial rates of pNPB hydrolysis, two values of rate constants (*k*_cat_) were determined corresponding to the first or second phase of the chemical process, depending on substrate concentrations. However, similar rate values for both stages (*k*_cat_^1^ of 0.41 ± 0.03 s^–1^ and *k*_cat_^2^ of 0.56 ± 0.01 s^–1^)^50^ suggest that the lid conformation does not significantly influence the chemical transformation steps taking place after diffusion of the substrate to the active site. Therefore, it can be inferred that as long as the substrate has access to the binding site of the enzyme, the reaction can proceed without a significant dependence on the degree of lid opening. Thus, to mimic the RCL efficiency with substrate low concentration, the quasi-open model of the protein can be selected for the studies of the chemical steps. Additional MD simulations on the protein:substrate noncovalent reactant complex were performed in order to select a representative structure of the reactive conformations for the subsequent exploration of the chemical steps of the catalyzed processes, esterase and urethanase activities of RCL, as described in the next sections.

### Esterase Activity of RCL

MD simulations carried out in 3 replicas of 500 ns each, confirmed the stable position of the docked pNPB in the active site with the preserved contact between Ser172 and carbonyl carbon (C1) (3.2 ± 0.1 Å) and a favorable Bürgi–Dunitz, α_BD_ angle, (defined as OG_S172_–C1_pNPB_–O1_pNPB_ of 80 ± 5°) as shown in Figure S8A, both ensuring serine ideal position for nucleophilic attack. Additionally, simulations confirmed the existence of two highly conserved hydrogen bonds established between carbonyl oxygen (O1) and oxyanion hole formed by the backbone −NH groups of Thr110 (2.2 ± 0.1 Å) and Leu173 (2.8 ± 0.1 Å). As expected, the presence of the substrate did not perturb the hydrogen bond interaction network established between Ser172, His284, and Asp231 in the catalytic triad. As shown in Figure 5A, the quasi-open conformation of the active site evolved in an explicit water solvent model with pNPB posed in the active site, with the Phe113 side chain adapting two orientations, the outward-facing conformation (characterized by the dihedral, φ_Phe113_ angle of −68°) dominating in 65% of all explored structures throughout the simulations. Less populated conformation (present in 32% of cases) corresponds to an inward orientation. This result demonstrates that in polar solution, the quasi-open conformation is maintained once the substrate enters the active site. The lid closure is compromised by the presence of the substrate, as shown in Figure 5 (part of the substrate occupies the original position of Phe113).

**Figure 5:**
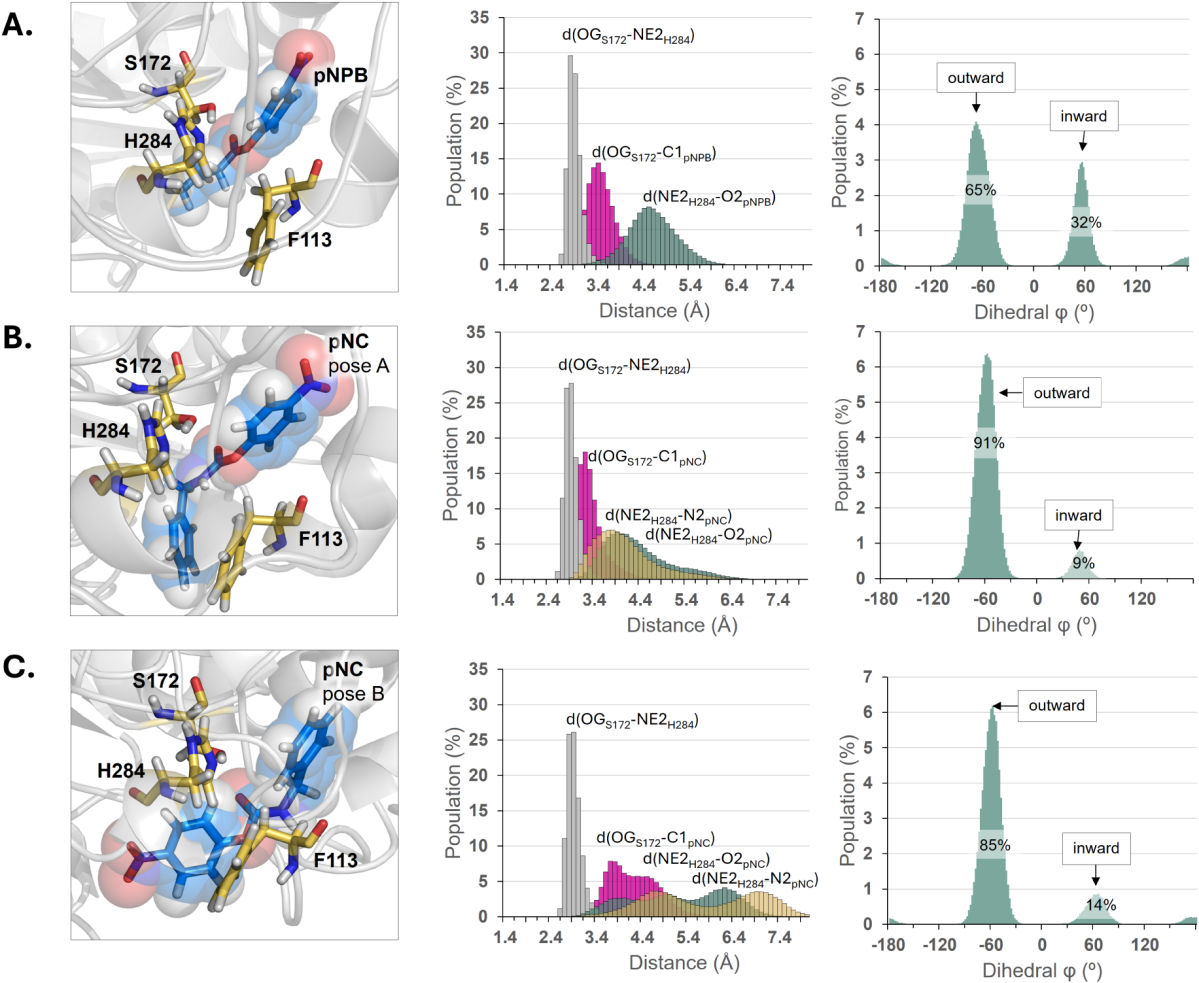
Structural analysis of the protein:substrate complex in the reactant state in the three studied systems: A. pNPB; B. pNC in Pose A; and C. pNC in Pose B. The first column shows a detail of the structure of the active sites, while the second and third columns show population analysis of key distances established between substrates and catalytic triad, and orientation of Phe113 residue, respectively. Results obtained for 150,000 snapshots generated during (3 × 500 ns) MD simulations.

To investigate the mechanism of pNPB hydrolysis catalyzed by RCL, the initial structure selected for calculation was a representative snapshot of the most populated cluster. The proposed mechanism was explored according to the previously described general mechanism for serine hydrolases, as illustrated in Figure 1.^80^ PESs were computed by supervising distances directly involved in the reaction progress, as detailed in the Supporting Information, followed by the generation of the FESs of all the chemical steps, as described in the Methods section. The obtained mechanism was confirmed by the optimization of four TS structures at the M06-2X/MM level and subsequently tracing the IRC paths. All the FESs for the reaction steps described hereafter are deposited in the Supporting Information (Figures S15–S17), as well as key interatomic distances obtained at the M06-2X/MM level for optimized structures of all states (Tables S5–S8). Schemes of the resulting reaction mechanisms, together with their corresponding free energy profiles, are shown in Figure 6. As expected, and in agreement with previous studies on other hydrolases,^43,45^ the reaction involves two chemical steps, the acylation of the substrate followed by the deacylation or hydrolysis step, both taking place in a stepwise manner.

**Figure 6:**
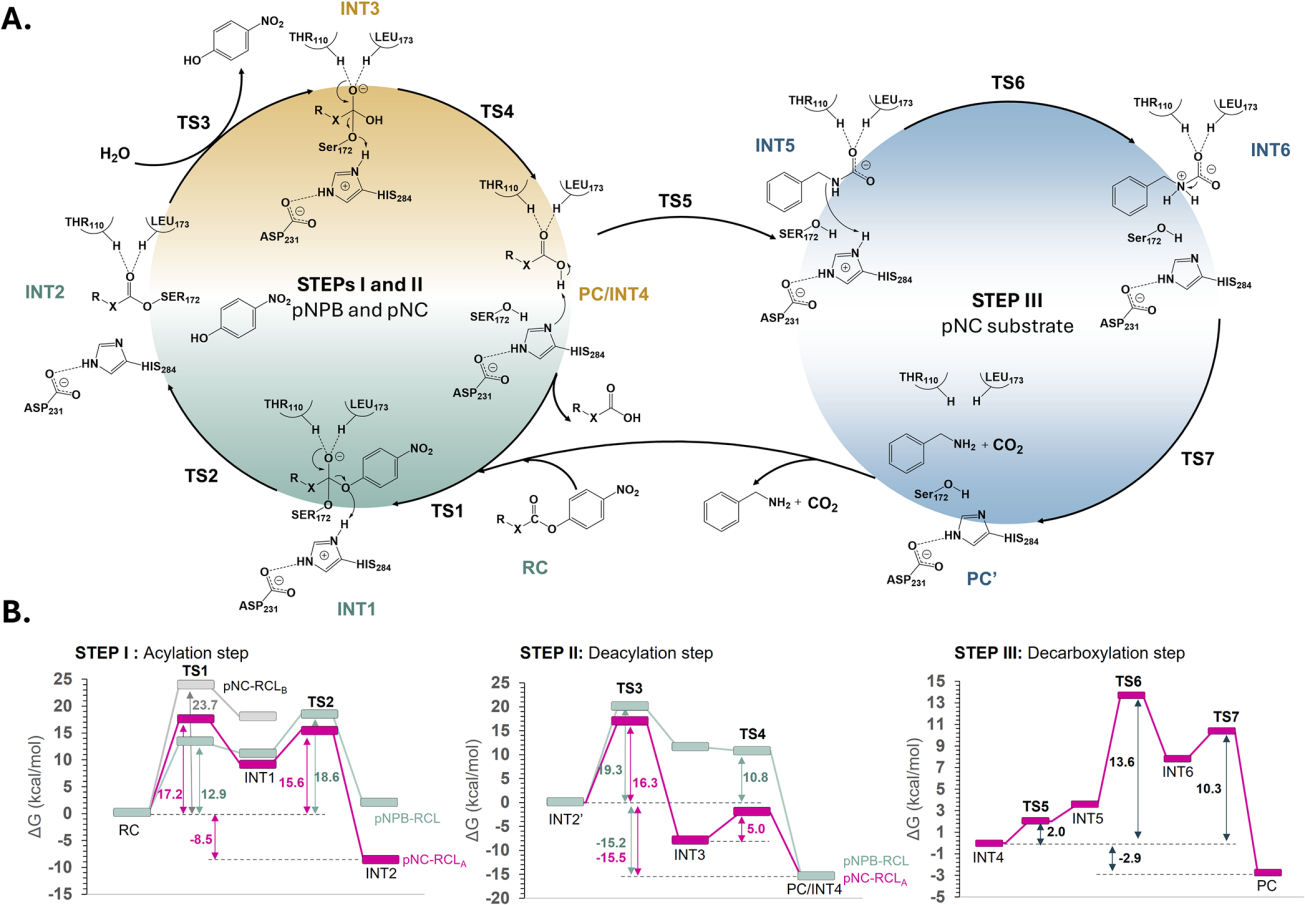
Computationally predicted esterase-like hydrolysis of the pNPB (STEP I and II) and pNC (STEP I, II, and III) substrates catalyzed by RCL. A. Scheme of the molecular mechanism. X is equal to C or N in the case of pNPB or pNC, respectively. B. M06-2X:AM1/AMBER free energy profiles for the esterase activity of RCL on pNPB (in gray) and pNC (in pink) computed for three steps: acylation, deacylation, and decarboxylation process (for pNC). Acylation of pNC was explored with two poses, Pose A and Pose B (see text for details). Values of reported free energies include ZPE corrections.

According to the free energy profile computed for the full chemical process with pNPB substrate, the second step of acylation and the first step of hydrolysis display equivalent barriers (18.6 and 19.3 kcal/mol, respectively), considering just the statistical uncertainty from MD samplings (ca. 1 kcal/mol) associated with the employed computational approach.^104–106^ These values are in good agreement with the activation free energies of Δ*G* ^‡^ of ∼18.8 kcal/mol derived from experimentally determined rate constants (*k*_cat_ of 0.45 ± 0.03 s^–1^,^47^ and 0.41 ± 0.03 s^–1^ and 0.43 ± 0.02 s^–1^ measured for RCL from *E. coli* and *P. Pastoris* measured at pH 8.5 and temperature of 40 °C,^51^ respectively) by applying the Transition State Theory (TST).^107^ Based on the deduced free energy profile, it was also found that the acylation step was slightly endergonic, with intermediate 2 (INT2) being 1.8 kcal/mol less stable than the reactant complex (RC). However, the final product of the hydrolysis reaction was found to be highly exergonic (Δ*G* = −15.2 kcal/mol), indicating that the process is strongly favored from a thermodynamic perspective. The excellent agreement between computational and experimental kinetic data supports the validity of the quasi-open RCL model employed in this study and reinforces the reliability of the computational methodology used to predict RCL activity toward the degradation of polyurethane samples, specifically the selected pNC model compound.

### Polyurethanase (PURase) Activity

PURase activity was investigated based on two possible binding poses of pNC within the active site, differing in the orientation of the p-nitrophenol moiety: directed inward the binding cavity (Pose A, pNC-RCL_A_) or outward (Pose B, pNC-RCL_B_), as discussed above and illustrated in Figure 5. Both orientations suggest reactive conformations in which the carbonyl carbon (C1) of pNC is positioned close to the oxygen atom (OG) of the catalytic Ser172, which would facilitate the nucleophilic attack in the initial step of the reaction mechanism, and the carbonyl oxygen atom pointing to the putative oxyanion hole formed by the backbone hydrogen atoms of residues Leu173 and Thr110. However, analysis of the two initial structures shows that the relative positioning of the NE2 nitrogen atom of His284 with respect to the N2 and O2 atoms of the substrate differs between Pose A and Pose B, suggesting the possibility of different reactivities of RCL. This is based on the likely proton transfer to the nearest atom of the substrate during the second step of the acylation, which is associated with the cleavage of the substrate bond, C–O or N–O. Thus, Pose A appears a priori more favorable for amidase activity, whereas Pose B suggests a potential esterase functionality. Three replicas of 500 ns NPT MD simulations were carried out for both pNC orientations, i.e., pNC-RCL_A_ and pNC-RCL_B_, to get more robust average structures. Geometrical analysis of the structures generated during simulations of pNC-RCL_A_ reveals the stability of Pose A, supported by the preservation of key interatomic distances essential for reaction initiation, as shown in Figure 5. In contrast, the alternative Pose B of pNC exhibited a wider distribution of those distances, indicating a less stable binding pose of PUR-like substrate in this orientation (Figure S8B,C). Moreover, analysis of the initial step of the acylation process revealed that the reaction is feasible only for the pNC-RCL_A_ complex, as indicated by a significantly lower free energy barrier calculated for TS1. As shown in Figure 6, the nucleophilic attack proceeds with an energy barrier of 17.2 kcal/mol when the substrate adopts Pose A, whereas in Pose B, the high barrier of 23.7 kcal/mol renders the process less favorable, which led us to focus exclusively on pNC-RCL_A_ for the subsequent steps.

The higher free energy barrier observed for Pose B can first be attributed to the lack of stabilization of the negative charge that accumulates on the O1 oxygen atom of the carbonyl group during the chemical transformation. This is likely due to the significantly greater distance between the backbone hydrogen of the oxyanion hole residue Thr110 and O1 in the TS1 structure, 3.64 Å in pose B, compared to 2.23 Å in Pose A, a trend also observed in the INT1 structure (3.98 Å in Pose B vs 2.14 Å in Pose A, see Tables S6 and S8 for details). Only interactions between O1 and Leu173 are not affected in both poses, including TS1 and INT1 structures (2.08 and 2.02 Å for TS1; 1.96 and 1.93 Å for INT1 in Poses A and B, respectively). Another reason for the barrier increment can be related to the different polarization of the nucleophilic OG_Ser172_ and C1_pNC_ in the reactant complex. Pose B shows a smaller atomic partial charge on OG_Ser172_and C1_pNC_ (−0.656 and 0.865 e^–^, respectively) compared to Pose A (−0.764 and 1.006 e^–^, respectively), indicating reduced nucleophilicity in Pose B (see Table S9). Furthermore, as demonstrated from MD simulations in Pose B, the electrophilic center position in RC is located further from the OG nucleophile atom (3.7 vs 3.2 Å for Poses B and A, respectively). This suggests that additional energy is required in Pose B to bring the reacting atoms into proximity for the reaction to occur. Based on the population analysis of key protein:substrate interatomic distances, Pose A appears initially suitable for supporting either amidase or esterase activity of RCL. This is particularly evident when considering the distances of 3.9 ± 0.1 Å and 3.7 ± 0.1 Å, respectively, measured in the noncovalent RC between the NE2 atom of the catalytic His284 (proton donor in the second step of acylation) and the O2/N2 atom of the carbamate linkage. We explored the viability of both possible mechanisms starting from the first intermediate (INT1), as shown in Figure 7. Exploration of the second step in the amidase pathway reveals that the reaction proceeds in a stepwise manner, initially forming a zwitterionic species (INT2^N^) before breaking the N2–C1 bond to release the benzylamine leaving group. It must be pointed out, however, that this zwitterionic intermediate is not relevant since it does not appear as a local minimum on the free energy landscape after adding ZPE corrections, despite being a local minimum in the potential energy surface, with lower energy than the preceding TS2^N^. The rate-limiting step for the stepwise process, measured from INT1 to the products of the amidase path INT3^N^, is defined by a low energy TS3^N^ (14.4 kcal/mol). However, it is revealed to be thermodynamically unfavorable due to a high free energy of the final product of the acylation step, i.e., 13.4 kcal/mol higher than the energy of RC. On the contrary, the esterase pathway proceeds with a relatively low barrier in the second acylation step (15.6 kcal/mol), resulting in an intermediate (INT2, product of the acylation) that is by 8.5 kcal/mol more stable than the RC and by 21.9 kcal/mol more stable than the product of amidase activity. Although local structural factors, such as substrate reorientation and the rearrangement of active-site water molecules, can influence the stabilization of intermediates along the reaction pathway, such perturbations are unlikely to compensate for the 13.4 kcal/mol energy difference relative to RC. This is particularly the case given that our free-energy profiles are based on QM/MM MD simulations that explicitly sample conformational space along the reaction coordinate. In this context, the preference for ester hydrolysis is governed primarily by the relative stability of the acylation products rather than by kinetic arguments. Thus, the lack of a stable amidase product indicates that this pathway is thermodynamically disfavored (see Table S11 of the Supporting Information).

**Figure 7:**
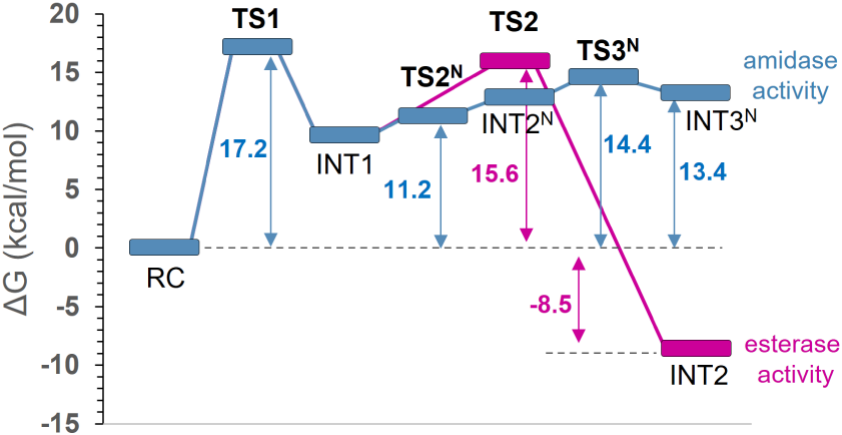
M06-2X/MM free energy profiles computed for the amino bond (blue line) and ester bond (pink line) cleavage of pNC taking place during the acylation step of the reaction catalyzed by RCL. Reported free energy values include ZPE corrections.

Given these results, further investigation into amidase activity was not pursued. The lack of stabilization of the acylation product in the case of C–N bond cleavage is not due to any dramatic change in the interactions between the products and the protein, as shown in Table S10 of the Supporting Information. Instead, it results from the inherent nature of the formed molecules, whose internal energy in this configuration is significantly higher than the products formed by ester bond cleavage. This is confirmed by potential energy calculations of the single structure of the QM subset of atoms in RC and both products in the gas phase. It was shown that INT2, the acylation product of esterase activity, was slightly less favorable than the reactant complex (by 0.6 kcal/mol), whereas INT3^N^, the product of amidase activity, was significantly less favorable, with an energy difference of 17.7 kcal/mol. A comparable energetic trend is observed in the QM/MM free energy profiles (Figure 7, Table S12 of the Supporting Information).

Subsequent investigation of the hydrolysis steps in the esterase pathway revealed that the reaction proceeds with an energy barrier of 16.3 kcal/mol. The overall hydrolysis reaction is exergonic, with the product complex exhibiting enhanced stability by 15.5 kcal/mol, as shown in Figure 6.

It is well-known that the benzylcarbamic acid formed as a product of the deacylation step (INT4) is thermally unstable in aqueous solution. The elusive character of carbamic acid derivatives originates from their tendency to lose carbon dioxide, thus reverting to the amine.^108^ However, we explored whether the decarboxylation process can be catalyzed in the active site of RCL, thus completing the hydrolysis of the substrate, until a product that could be observed experimentally was achieved. In this study, we assumed active participation of His284 in this process, which could facilitate proton rearrangement and CO_2_ departure. As shown in Figure 6 and confirmed by three optimized TSs, decarboxylation consists of three steps. These steps involve: proton abstraction from the carboxyl group by His284, leading to the formation of an ionic pair (INT5); transfer of the proton to the amino group, resulting in a zwitterionic intermediate (INT6); and, finally, the breaking of the C1–N2 bond, which allows for the release of benzylamine and CO_2_ molecules. The computed free energy profile, provided in Figure 6, revealed a feasible and mildly exergonic process, with Δ*G*_r_ of −2.9 kcal/mol, proceeding with a relatively low overall activation free energy of 13.6 kcal/mol. Analogously to what was observed for the amidase pathway, the nature of the chemical step and the small energy differences between the involved states cause INT5 to appear slightly higher than the preceding TS5 in the vibrational corrected free energy profile, even though TS5 was characterized as a first-order saddle point on the potential energy surface (see Table S12 in the Supporting Information). As in the INT1 to INT2^N^ step discussed above, this feature has not kinetic relevance for the stepwise decarboxylation process. Notably, the rate constant for the spontaneous decarboxylation of benzylcarbamic acid in solution has been reported as 49 s^–1^ at 25 °C,^109^ corresponding to an activation free energy of 15.1 kcal/mol based on TST. Therefore, as shown here, decarboxylation can be accelerated with the assistance of the RCL active site since our simulations predict a rate constant of 2.1 × 10^3^ s^–1^ by applying a higher temperature (40 °C). Based on these results, it can be concluded that the decarboxylation process does not influence the overall rate of RCL PURase activity, indicating that its impact on the reaction speed is negligible.

### Comparative Analysis of Esterase and PURase Activities

Analysis of the obtained reaction mechanisms and the associated free energy profiles reveals significant differences depending on the explored substrate despite that, in both cases, the ester (C1_Sub_–O2_Sub_) bond is broken during the acylation step. The rate-determining step in the acylation stage for pNPB is the second step, when the ester bond scission takes place (18.6 kcal/mol), while for pNC, the nucleophilic attack is the rate-determining step (17.2 kcal/mol). The larger energy required to break the C–O bond in pNPB can be partially attributed to the weaker stabilization of INT1 within the active site, by 2.1 kcal/mol compared to pNC, which contributes to the overall TS2 barrier. Charge analysis of representative structures optimized at M06-2X/MM shows that in INT1 of the pNPB degradation process, the ester bond was found to be less polarized than in INT1 of pNC, as indicated by the lower positive charge on the C1_Sub_ carbon atom and virtually the same charge on O2_Sub_ oxygen atom (Table 1). This slightly reduced polarization is in agreement with a slightly higher energy barrier for bond dissociation (from 6.4 to 7.3 kcal/mol as computed for pNC and pNPB, respectively).

**Table 1:** Charges from Electrostatic Potentials Using a Grid-Based Method (CHelpG) Provided in Atomic Units (e^–^), Computed for Key Atoms for Optimized Structures at the M062X/AMBER Level along the Acylation (RC to INT1) and Deacylation (I2 to INT3) Steps

			C1_Sub_	O2_Sub_/O_wat_	O1_Sub_	N2_pNC_	OG_Ser172_	NE2_His284_
**RC → INT1**	pNC	RC	1.006	–0.611	–0.705	–0.616	–0.764	–0.241
		TS1	1.046	–0.564	–0.802	–0.602	–0.662	–0.125
		INT1	1.113	–0.640	–0.903	–0.611	–0.645	0.042
	pNPB	RC	0.975	–0.577	–0.689	–	–0.788	–0.157
		TS1	1.058	–0.619	–0.862	–	–0.803	–0.026
		INT1	1.056	–0.641	–0.918	–	–0.797	–0.007
**INT2 → INT3**	pNC	INT2	0.690	–0.795	–0.668	–0.163	–0.607	–0.351
		TS3	1.063	–0.912	–0.868	–0.591	–0.731	–0.052
		INT3	0.931	–0.672	–0.961	–0.454	–0.667	0.166
	pNPB	INT2	0.821	–0.745	–0.671	–	–0.710	–0.131
		TS3	0.854	–0.606	–0.862	–	–0.750	–0.009
		INT3	0.828	–0.646	–0.979		–0.769	0.119

However, larger differences are observed on the first step of the acylation between the two reactions, 4.3 kcal/mol higher for the pNC substrate compared with the corresponding step in the alternative reaction. These differences can be rationalized by the nature of TS1. The nucleophilic attack and proton transfer take place in a strictly concerted manner in pNC, with a 1.76 Å C1_Sub_–OG_Ser172_ distance, and a proton placed at equal distances between the donor (OG_Ser172_–HG_Ser172_ of 1.23 Å) and acceptor (NE2_His284_–HG_Ser172_ of 1.29 Å) atoms. In contrast, in the TS1 for the corresponding chemical step with pNPB, although the distance between the nucleophile and the C1 atom is similar to the one observed in pNC (C1_Sub_–OG_Ser172_ of 1.71 Å), a more advanced proton transfer was identified, with the hydrogen placed closer to its acceptor (NE2_His284_–HG_Ser172_ of 1.18 Å). This shift leads to a significant increase in the negative charge on the nucleophilic OG atom of Ser172, from −0.662 e^–^ in the TS of pNC to −0.803 e^–^ in pNPB. Thus, the slightly higher charge change on this atom from RC to TS1 in pNC than in pNPB, (−0.102 vs 0.015 e^–^) is in agreement with a larger energy barrier.

Similar conclusions can be derived when comparing the first step of the deacylation for esterase and PURase activities. As shown in Figure 6, the water attack on the acyl group is more energetically demanding in the case of pNPB than in the case of pNC, with computed barriers of 19.3 and 16.3 kcal/mol, respectively. Geometrical analysis of the TS3 localized for both substrates revealed similar distances between the oxygen of water and the carbon of the substrate (1.72 Å in pNPB and 1.73 Å in pNC). However, again, a significant difference was observed in the position of the transferred proton. In pNPB, the hydrogen of the water molecule is only partially dissociated from the oxygen (O_Wat_–H1_Wat_ of 1.13 Å), whereas in pNC, the water molecule appears more deprotonated (O_Wat_–H1_Wat_ of 1.37 Å). These data show that the water oxygen in TS3 bears a significantly more negative charge in the pNC system (−0.912 e^–^) than in the corresponding pNPB (−0.606 e^–^). Considering the charges in the preceding INT2 minima (−0.795 e^–^ and −0.745 e^–^, respectively), a slightly smaller charge rearrangement is observed in this step for pNC than for pNPB, in line with the lower activation barrier computed for the former. More importantly, while the negative charge increases from INT2 to TS3 in the pNC pathway, it decreases in the case of pNPB. When this evolution of atomic charges is combined with the positive electrostatic potential exerted by RCL in the active site across all reaction states (approximately +300 to +500 kJ·mol^–1^·e^–1^; see Table S13 in the Supporting Information), it follows that TS3 is more electrostatically stabilized in the pNC system than in pNPB.

Overall, the observed trends in substrate charge polarization, together with the positive electrostatic potential generated by RCL in the active site, correlate well with the differences in the calculated activation barriers.

## Conclusions

In this study, we investigated the hydrolysis of a polyurethane (PUR) model compound, 4-nitrophenyl benzylcarbamate (pNC), catalyzed by *Rhizopus chinensis* lipase (RCL) to evaluate its potential for cleaving urethane bonds in polyurethane plastics. We first explored the conformational landscape of the apoenzyme through GBIS MD simulations in water and hexane. In aqueous environments and low substrate concentrations, RCL adopts a closed conformation that restricts access to the active site. Activation of the enzyme requires conformational changes involving not only the previously proposed rotation of Phe113 but also a significant rearrangement of Arg114. This can be achieved by the use of nonpolar solvents or a high solute concentration. Thus, upon substrate accumulation, strong electrostatic interactions between Arg114 and Asp11/Glu3 are disrupted, enabling Arg114 to reorient toward Phe113. This movement induces the outward shift of Phe113, opening a channel for substrate entry, even with the lid adopting a closed conformation. MD simulations confirmed this conformational transition and revealed stable substrate binding near Ser172 during the MD trajectory. Results derived from additional MD simulations constraining the initial orientation of Arg114 support the impact of the reorientation of this residue in opening the access to the active site. The classical interfacial activation mechanism of lipases is revisited in light of these findings. From a computational perspective, the results support the use of a quasi-open RCL conformation for modeling its catalytic activity with small compounds such as *p*-nitrophenyl butyrate (pNPB) and pNC.

RCL’s activity was first benchmarked against pNPB, a standard lipase substrate with available experimental kinetic data, by exploring the conventional two-step process: acylation and hydrolysis. The computed M06-2X/MM free energy barriers for the second step of the acylation and the first step of the hydrolysis (18.6 and 19.3 kcal/mol, respectively) align well with experimental values (18.8 kcal/mol),^47,50^ validating our simulation protocol and providing a reference point for comparison with the RCL’s activity on the polyurethane model compound.

The analysis of the binding and reactivity of the asymmetric pNC compound reveals two possible orientations within the active site with virtually the same favorable interaction energies: Pose A, with the nitrophenyl group oriented inward, and Pose B, oriented outward. Pose A was found to be catalytically favorable, enabling proper nucleophilic alignment and lower free energy barriers. Pose B presents an increased dispersion in key geometrical parameters or broader conformational distributions. Consequently, Pose B showed a higher energy barrier for nucleophilic attack (23.7 vs 17.2 kcal/mol in Pose A). From this analysis, C–N or C–O breaking bonds was explored in the acylation step. The results show how, despite Pose A supporting amidase activity, the pathway was thermodynamically unfavorable, with a product of the acylation step 13.4 kcal/mol higher than the reactant complex. Conversely, the acylation step in the esterase pathway leads to a state (INT2) 8.5 kcal/mol more stable than the initial RC. From this intermediate, the reaction proceeds via a deacylation (hydrolysis) step that is thermodynamically favorable (Δ*G* = −15.5 kcal/mol) and kinetically feasible (Δ*G* ^‡^ = 16.3 kcal/mol), producing benzylcarbamic acid as the final product of the hydrolysis stage.

Our simulations show how decarboxylation of this compound to benzylamine and CO_2_ via a three-step mechanism is feasible within the enzyme active site and does not limit the overall hydrolysis rate, considering the overall activation barrier (13.6 kcal/mol) and exergonic nature (Δ*G* = −2.9 kcal/mol) of this step. This decarboxylation, catalyzed inside the active site of the protein, can compete with the corresponding spontaneous process in solution, supporting RCL’s potential to catalyze the full degradation of pNC. Although the hydrolysis of pNPB and pNC proceeds through similar mechanisms, noticeable energetic differences are observed. These differences can be rationalized, at least in part, by analyzing the evolution of atomic charges and the electrostatic potential generated by RCL within the active site. The greater polarization and nucleophilicity of pNC compared with pNPB, together with a robust positive electrostatic potential created by RCL in the active site, are consistent with the computed activation free energies.

Altogether, our data show that RCL acts predominantly via esterase activity on the pNC model and is slightly more efficient in degrading the selected PUR-like compound than a conventional ester substrate. This is due to more favorable electrostatics and geometries in the transition states, including not only optimal residue positioning but also long-range electrostatic effects that help stabilize the charge redistribution during bond cleavage and formation.

Our results suggest that RCL can fully hydrolyze the PUR model compound in aqueous media, as long as a locally nonpolar microenvironment is achieved by high concentration of substrate or formation of micelles, in agreement with previous experimental evidence,^50,55^ and provide a detailed atomistic framework for understanding RCL’s polyurethanase activity. Interestingly, the computed free energy barriers indicate that RCL may exhibit greater catalytic efficiency in hydrolyzing the selected PUR model compound than UMG-SP2, originally discovered by Bornscheuer and coworkers,^30^ which, according to our previous study, hydrolyzes the same compound at a lower rate constant (2.06 x10^–4^ s^–1^).^45^ Increasing the population of the open-lid conformation may further enhance catalytic efficiency. However, as large polymer chains cannot fit into the binding pocket in the quasi-open conformation, a fully open lid may be necessary for real plastic depolymerization, which can be achieved by changing the solvent, as observed in the hexane GBIS MD simulations, or by designed protein mutants. As with any computational prediction, our hypothesis will require future experimental validation, which remains the cornerstone of scientific progress. In all, this work represents a foundational step toward the development of efficient RCL-based PURases for biotechnological applications in polyurethane recycling.

## Supplementary Material





## Data Availability

As of now, no standard database for MD data exists (see ref^110^ for a discussion); reduced trajectories (3,000 snapshots representing 300 ns of GBIS simulation and 5,000 snapshots of each 500 ns from MD simulations with an explicit solvent model) have been deposited at Zenodo: DOI: https://10.5281/zenodo.

## Notes

Movie S1: Spontaneous substrate binding mechanism constructed from 500 ns of unbiased MD simulations of RCL in a simulated micellar concentration of pNPBs (MP4)
Structural analysis of similar 3D structures to RCL; docking results; prediction of p*K*_a_ data of titratable residues; force field parameters for pNPB and pNC; p*K*_a_ values of titratable residues computed with PROPKA3.1; time-dependent evolution and population analysis of MD trajectories; protein–substrate interaction energies. Details of the active site and QM-MM partitioning and extended computational details; M06-2X/6-31+G­(d,p):AM1/MM FESs of every chemical step; key distances of structures optimized at M06-2X:AM1/AMBER level; window overlap analysis for the PMFs of every FES; charges from electrostatic potentials using a grid-based method (CHelpG); average electrostatic potential generated by the protein computed on the key atoms of the active site of RCL; coordinates of QM atoms of structures of transition states optimized at M06-2X/6-31+G­(d,p)/AMBER level; complete structures are available from the authors upon request (PDF)
