# Supplementary material for: From Brewing to Plastic Degradation: Uncovering the Polyurethanase Potential of R. chinensis Lipase through Atomistic Simulations
Source: J Am Chem Soc. 2026 Feb 19;148(8):8795–811. doi: 10.1021/jacs.5c21421 (PMC12964413; doi:10.1021/jacs.5c21421)
Supplement: Supplementary file 2 [file ja5c21421_si_002.pdf]

Supporting Information

From Brewing to Plastic Degradation: Uncovering the Polyurethanase Potential of *R. chinensis* Lipase through Atomistic Simulations

Victor de Sousa Batista, Katarzyna Świderek and Vicent Moliner**

BioComp Group, Institute of Advanced Materials (INAM), Universitat Jaume I, 12071 Castellón, Spain.

---

1. System setup......3

Structures of macromolecules with similar 3-dimensional structures to RCL......3

Representation of the open and closed lid conformations......4

Selected binding pose of pNPB obtained with Autodock Vina......5

Predicted pKa values of the titratable residues of RCL......6

FF parameters obtained for pNPB substrate......7

FF parameters obtained for pNC substrate......9

2. MD Simulations......11

Time-dependent evolution of the RMSD for protein and substrate heavy atoms......11

Time-dependent evolution of key distances and angles in the E:S of the pNPB-RCL model...12

Time-dependent evolution of key distances and angles in the E:S of the pNC-RCL$_A$ model....13

Time-dependent evolution of key distances and angles in E:S of the pNC-RCL$_B$ model......14

Population analysis for the Bürgi-Dunitz angle and for the distances established for substrates and oxyanion hole......15

Time-dependent evolution and population analysis of Phe113 side chain conformers......16

Protein-Substrate interaction energies......17

3. QM/MM simulations......19

Definition of QM region in the active site of RCL......19

Detailed information on the exploration of individual potential energy surfaces (PES)......20

Free energy surfaces computed for all chemical steps calculated for the pNPB-RCL system22

Free energy surfaces computed for all chemical steps calculated for the pNC-RCL$_A$ system.23

Free energy surfaces computed for all chemical steps calculated for the pNC-RCL$_B$ system..24

PMF window overlap analysis......24

Key distances for the structures optimized along the degradation of pNPB......26

Key distances for the structures optimized along the degradation of pNC$_A$ via esterase activity......27

Key distances for the structures optimized along the degradation of pNC$_A$ via amidase activity......28

Key distances for the structures optimized along the degradation of pNC$_B$............................28
*CHarges from ELectrostatic Potentials using a Grid-based method for optimized structures*29
Interaction energy calculated for INT2(esterase) and INT3^N in system pNC-RCL$_A$................30
Potential energy calculated for INT2(esterase) and INT3^N of pNC-RCL$_A$ in vacuum............31
Free energy derived from the FELs...31
Electrostatic potential (V_{elec})...32
4. Cartesian coordinates of QM atoms for the optimized transition states. 33
5. References...42

1. System setup

Structures of macromolecules with similar 3-dimensional structures to RCL

Table S1. Structures of macromolecules with similar 3-dimensional structures to RCL, and significantly high RMSD values and a high number of aligned residues identified using VAST+ software.

	PDB ID	Taxonomy	RMSD	Aligned Residues	Sequence Identity	LID residues	LID overlay
**1**	5GW8	*Malassezia globosa CBS 7966*	2.34 Å	247	30%	80-96	NO
**2**	1DTE	*Thermomyces lanuginosus*	2.46 Å	247	31%	80-96	NO
**3**	3NGM	*Fusarium graminearum*	2.27 Å	255	31%	80-96	YES
**4**	3O0D	*Yarrowia lipolytica*	2.55 Å	253	31%	86-102	YES
**5**	1USW	*Aspergillus niger*	1.90 Å	236	32%	67-83	NO
**6**	5CH8	*Penicillium cyclopium*	2.31 Å	250	24%	80-96	YES

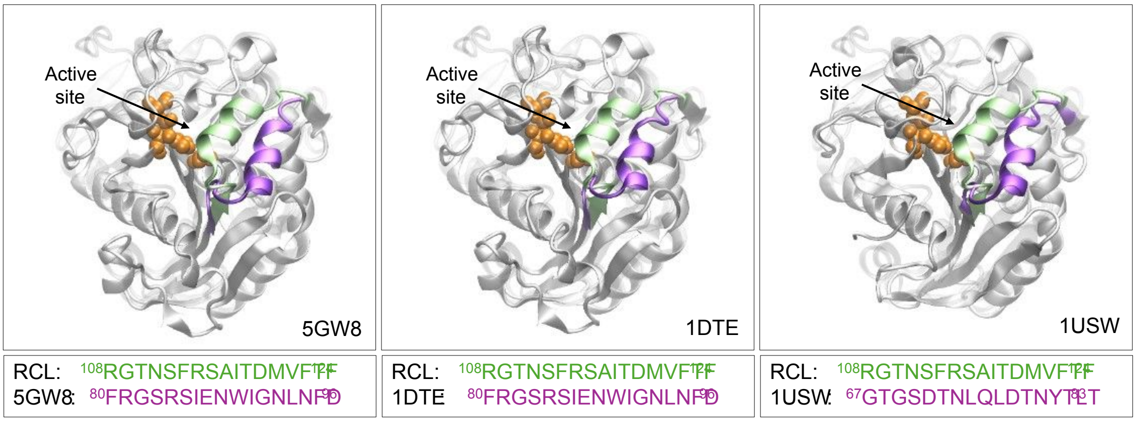

Figure S1. Overlay of RCL with lipases from *Malassezia globosa* (5GW8), *Thermomyces lanuginosus* (1DTE), and *Aspergillus niger* (1USW). The position of the closed lid of RCL is highlighted in green, while the position of the open-lid conformation is shown in purple.

Representation of the open and closed lid conformations

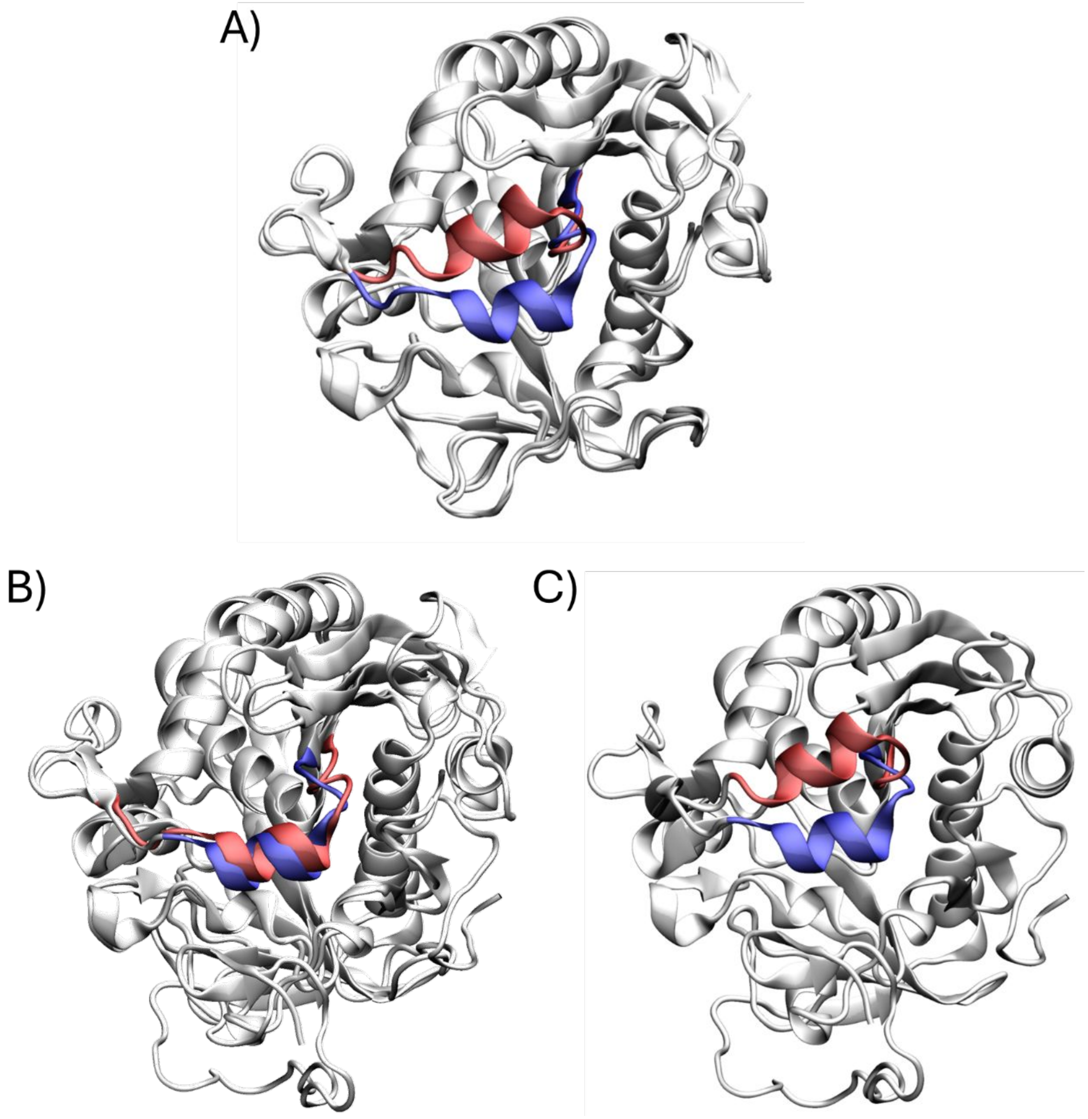

Figure S2. Representation of the open (red) and closed (blue) lid conformations experimentally observed for *Thermomyces lanuginosus* (TLL) (A). Overlay of the RCL (blue) with TLL (red) in the closed conformation. (B) Open conformation (red) produced *in silico* for RCL overlayed with the closed (blue) conformation experimentally observed (C).

Selected binding pose of pNPB obtained with Autodock Vina

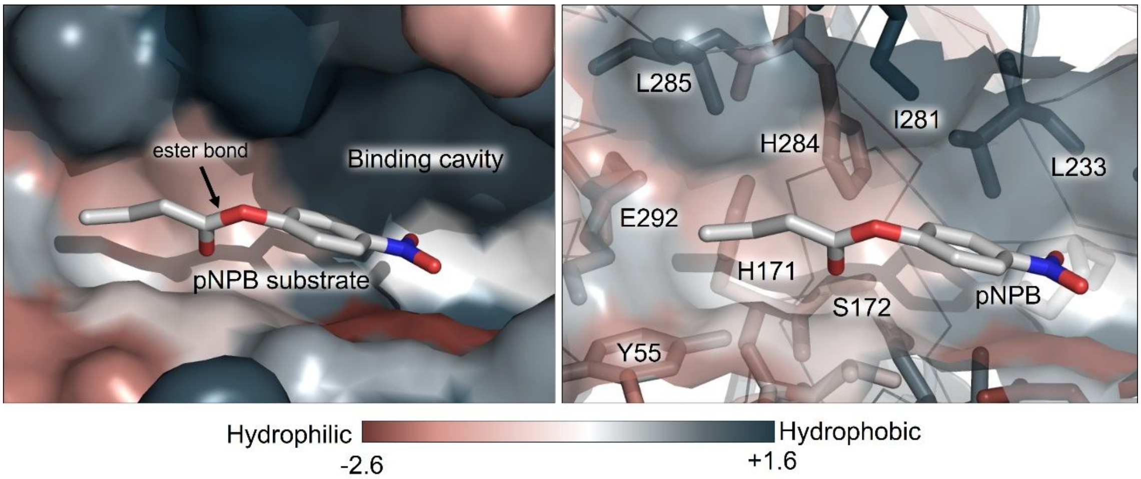

Figure S3. Selected binding pose of pNPB obtained with Autodock Vina used as the starting point for GBIS MD simulation. Hydrophobicity map generated based on the Eisenberg hydrophobicity scale.[1]

Predicted pKa values of the titratable residues of RCL

Table S2. Predicted pKa values of the titratable residues of RCL using the PROPKA version 3 program.

**aa**	**pK$_a$**	**aa**	**pK$_a$**	**aa**	**pK$_a$**
ASP11	2.43	HIS171	1.05	TYR287	17.34
ASP29	3.09	HIS224	4.21	LYS42	10.36
ASP66	3.81	HIS228	3.45	LYS64	10.56
ASP76	2.74	HIS235	3.21	LYS68	10.30
ASP89	3.37	HIS245	1.60	LYS72	11.11
ASP98	0.64	HIS284	1.02	LYS78	11.27
ASP119	3.74	HIS300	8.61	LYS81	10.22
ASP126	3.18	HIS301	5.92	LYS101	11.32
ASP149	4.57	HIS302	8.66	LYS131	10.47
ASP156	4.00	HIS303	6.20	LYS134	11.08
ASP163	2.63	HIS304	6.53	LYS148	10.42
ASP184	3.04	HIS305	6.71	LYS165	10.01
ASP217	6.19	CYS204	12.12	LYS190	10.39
ASP231	8.04	TYR47	12.26	LYS195	10.42
ASP255	3.16	TYR55	15.82	LYS229	10.46
ASP258	3.77	TYR73	12.32	LYS253	9.47
ASP283	1.94	TYR104	13.28	LYS269	10.28
GLU3	4.59	TYR127	12.77	ARG57	12.40
GLU14	3.63	TYR143	19.11	ARG96	14.26
GLU32	4.83	TYR150	14.00	ARG108	11.57
GLU43	4.89	TYR161	10.17	ARG114	13.64
GLU189	2.11	TYR164	12.11	ARG188	14.48
GLU249	5.58	TYR186	10.33	ARG191	14.04
GLU254	2.52	TYR200	13.89	ARG206	13.36
GLU267	0.25	TYR214	9.94	ARG225	11.48
GLU292	9.86	TYR215	11.06	ARG230	11.38
HIS136	3.98	TYR243	12.93		

FF parameters obtained for pNPB substrate

Table S3. Atom types, charges (in a. u.), and FF parameters obtained for pNPB substrates, at AM1-BCC level of theory and using GAFF2 force field.

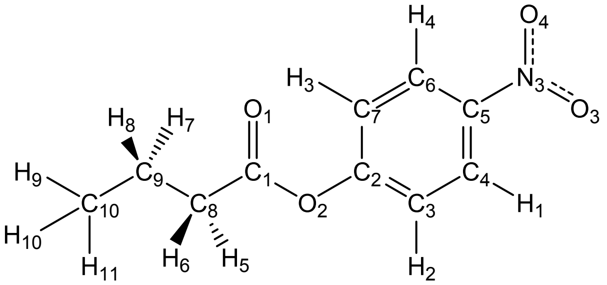

pNPB								
**Label**	**Type**	**Charge**	**Label**	**Type**	**Charge**	**Label**	**Type**	**Charge**
C5	ca	-0.0505	O2	os	-0.3732	H4	ha	0.1745
C4	ca	-0.1545	C1	c	0.6451	H5	hc	0.0852
C3	ca	0.1581	O1	o	-0.5100	H6	hc	0.0852
C2	ca	-0.1545	C8	c3	-0.1274	H7	hc	0.0582
C7	ca	-0.0505	C9	c3	-0.0804	H8	hc	0.0582
C6	ca	-0.1942	C10	c3	-0.0951	H9	hc	0.0400
N3	no	0.3162	H1	ha	0.1745	H10	hc	0.0400
O3	o	-0.2095	H2	ha	0.1675	H11	hc	0.0400
O4	o	-0.2095	H3	ha	0.1675			
**MASS**			**DIHEDRAL**					
ca	12.01	0.360	ca-ca-ca-no		4	14.50	180	2
ha	1.008	0.135	ca-ca-ca-ca		4	14.50	180	2
no	14.01	0.530	ca-ca-os-c		2	1.80	180	2
o	16.00	0.434	ha-ca-ca-ha		4	14.50	180	2
os	16.00	0.465	ca-ca-ca-ha		4	14.50	180	2
c	12.01	0.616	ha-ca-ca-os		4	14.50	180	2
c3	12.01	0.878	ca-ca-ca-os		4	14.50	180	2
hc	1.008	0.135	ca-ca-no-o		4	2.40	180	2
			ha-ca-ca-no		4	14.5	180	2
**BOND**			o-c-os-ca		2	5.40	180	2
ca-ha	345.8	1.086	c3-c-os-ca		2	5.40	180	2
ca-ca	461.1	1.398	os-c-c3-hc		6	0.0	180	2
ca-no	321.7	1.469	os-c-c3-c3		6	0.0	180	2
no-o	741.8	1.226	c-c3-c3-hc		9	1.40	0	3
ca-os	376.6	1.370	c-c3-c3-c3		9	1.40	0	3
c-os	390.8	1.358	o-c-c3-hc		1	0.80	0	-1
c-o	637.7	1.218	o-c-c3-hc		1	0.00	0	-2
c-c3	313.0	1.524	o-c-c3-hc		1	0.08	180	3
c3-hc	330.6	1.097	o-c-c3-c3		6	0.00	180	2
c3-c3	300.9	1.538	c3-c3-c3-hc		1	0.16	0	3
			hc-c3-c3-hc		1	0.15	0	3

**ANGLE**			**IMPROPER**					
ca-ca-ha	48.2	119.88	ca-ca-ca-ha		1.1	180	2	
ca-ca-ca	66.6	120.02	ca-ca-ca-no		1.1	180	2	
ca-ca-os	69.6	119.20	ca-o-no-o		1.1	180	2	
ca-ca-no	66.8	119.01	ca-ca-ca-os		1.1	180	2	
ca-no-o	68.7	117.76	c3-o-c-os		1.1	180	2	
o-no-o	76.7	125.08						
c-os-ca	63.4	121.15		**NONBOND**				
o-c-os	75.3	123.25		ca	1.908	0.0860		
c3-c-os	68.9	110.72		ha	1.459	0.0150		
c-c3-hc	46.9	108.77		no	1.824	0.1700		
c-c3-c3	63.3	111.04		o	1.6612	0.2100		
c3-c-o	67.4	123.20		os	1.6837	0.1700		
c3-c3-hc	46.3	109.80		c	1.908	0.0860		
c3-c3-c3	62.9	111.51		c3	1.908	0.1094		
hc-c3-hc	39.4	107.58		hc	1.487	0.0157		

FF parameters obtained for pNC substrate

Table S4. Atom types, charges (in a. u.) and FF parameters obtained for pNC substrate at AM1-BCC level of theory and using GAFF2 force field.

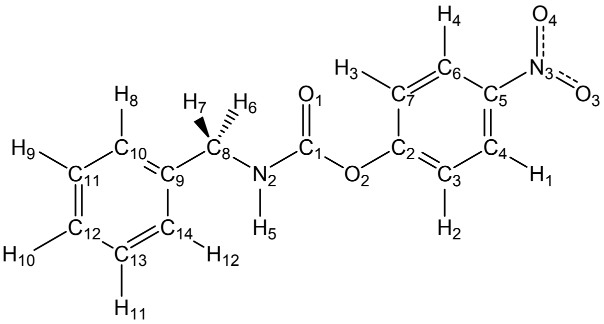

**pNC**								
**Label**	**Type**	**Charge**	**Label**	**Type**	**Charge**	**Label**	**Type**	**Charge**
C5	ca	-0.1910	C10	ca	-0.1140	H3	ha	0.170
C4	ca	-0.0540	C11	ca	-0.1290	H4	ha	0.175
C3	ca	-0.1490	C12	ca	-0.1170	H5	hn	0.317
C2	ca	0.1671	C13	ca	-0.1290	H6	h1	0.081
C7	ca	-0.1490	C14	ca	-0.1140	H7	h1	0.081
C6	ca	-0.0540	O1	o	-0.5560	H8	ha	0.136
O2	os	-0.3760	N3	no	0.3152	H9	ha	0.137
C1	c	0.7341	O3	o	-0.2100	H10	ha	0.136
N2	ns	-0.4980	O4	o	-0.2100	H11	ha	0.137
C8	c3	0.1203	H1	ha	0.1745	H12	ha	0.136
C9	ca	-0.1380	H2	ha	0.1695			
**MASS**			**DIHEDRAL**					
ca	12.00	0.360	ca-ca-ca-no		4	14.5	180	2
ha	1.01	0.135	ca-ca-ca-ca		4	14.5	180	2
no	14.00	0.530	ca-ca-os-c		2	1.8	180	2
o	16.00	0.434	ha-ca-ca-ha		4	14.5	180	2
os	16.00	0.465	ca-ca-ca-ha		4	14.5	180	2
c	12.00	0.616	ha-ca-ca-os		4	14.5	180	2
ns	14.00	0.530	ca-ca-ca-os		4	14.5	180	2
hn	1.01	0.161	ca-ca-no-o		4	2.4	180	2
c3	12.00	0.878	ha-ca-ca-no		4	14.5	180	2
h1	1.01	0.135	o-c-os-ca		2	5.4	180	2
			ns-c-os-ca		2	5.4	180	2
**BOND**			os-c-ns-hn		4	10	180	2
ca-ha	346	1.086	os-c-ns-c3		4	10	180	2
ca-ca	461	1.398	h1-c3-ns-c		6	0.0	0	2
ca-no	322	1.469	ca-c3-ns-c		6	0.0	0	2
no-o	742	1.226	o-c-ns-hn		1	2.5	180	-2
ca-os	377	1.370	o-c-ns-hn		1	2.0	0	1
c-os	391	1.358	o-c-ns-c3		4	10	180	2

c-o	638	1.218	ns-c3-ca-ca		6	0.0	0	2
c-ns	428	1.379	h1-c3-ns-hn		6	0.0	0	2
hn-ns	403	1.013	ca-c3-ns-hn		6	0.0	0	2
c3-ns	329	1.462	c3-ca-ca-ha		4	14.5	180	2
c3-h1	331	1.097	c3-ca-ca-ca		4	14.5	180	2
c3-ca	321	1.516	h1-c3-ca-ca		6	0.0	0	2
**ANGLE**			**IMPROPER**					
ca-ca-ha	48.2	119.88	ca-ca-ca-ha		1.1	180	2	
ca-ca-ca	66.6	120.02	ca-ca-ca-no		1.1	180	2	
ca-ca-os	69.6	119.20	ca-o-no-o		1.1	180	2	
ca-ca-no	66.8	119.01	ca-ca-ca-os		1.1	180	2	
ca-no-o	68.7	117.76	ns-o-c-os		1.1	180	2	
o-no-o	76.7	125.08	c-c3-ns-hn		1.1	180	2	
c-os-ca	63.4	121.15	c3-ca-ca-ca		1.1	180	2	
o-c-os	75.3	123.25						
ns-c-os	75.3	109.22		**NONBOND**				
c-ns-hn	48.3	117.55		ca	1.9080	0.09		
c-ns-c3	63.4	120.69		ha	1.4590	0.02		
ns-c-o	74.2	123.05		no	1.8240	0.17		
h1-c3-ns	49.8	108.88		o	1.6612	0.21		
ca-c3-ns	66.2	112.38		os	1.6837	0.17		
c3-ns-hn	45.8	117.68		c	1.9080	0.09		
c3-ca-ca	63.5	120.77		ns	1.8240	0.17		
h1-c3-h1	39.2	108.46		hn	0.6000	0.02		
ca-c3-h1	47.0	109.56		c3	1.9080	0.11		
				h1	1.3870	0.02		

2. MD Simulations

Time-dependent evolution of the RMSD for protein and substrate heavy atoms

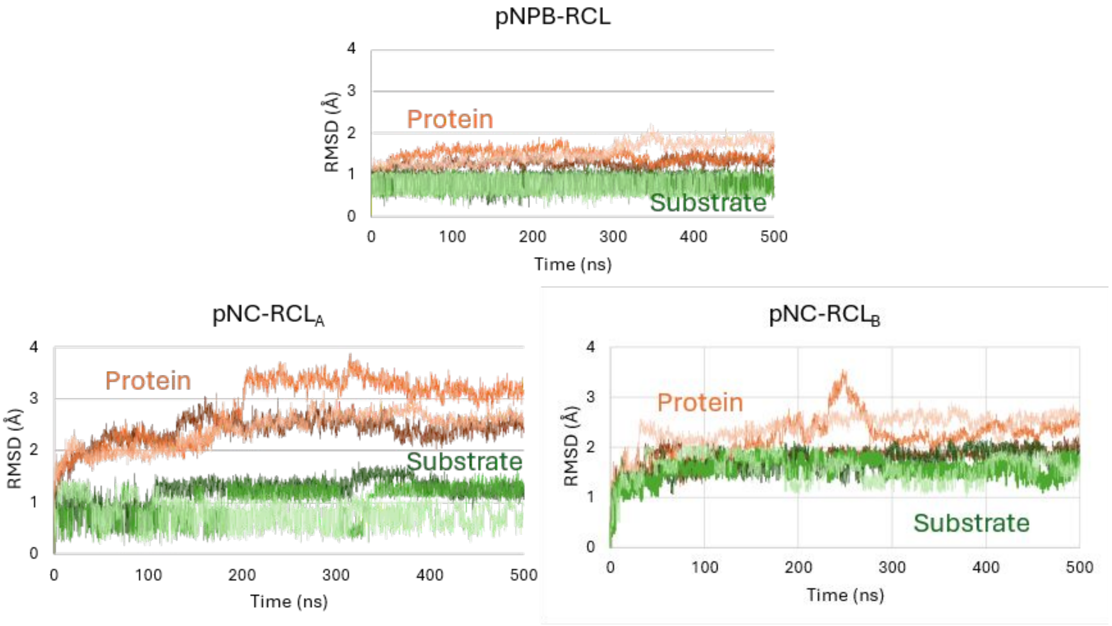

Figure S4. Time-dependent evolution of the RMSD computed with the backbone atoms (CA, N, C and O, in orange) and substrate heavy atoms (in green) for each protein:substrate model at the RC state.

Time-dependent evolution of key distances and angles in the E:S of the pNPB-RCL model

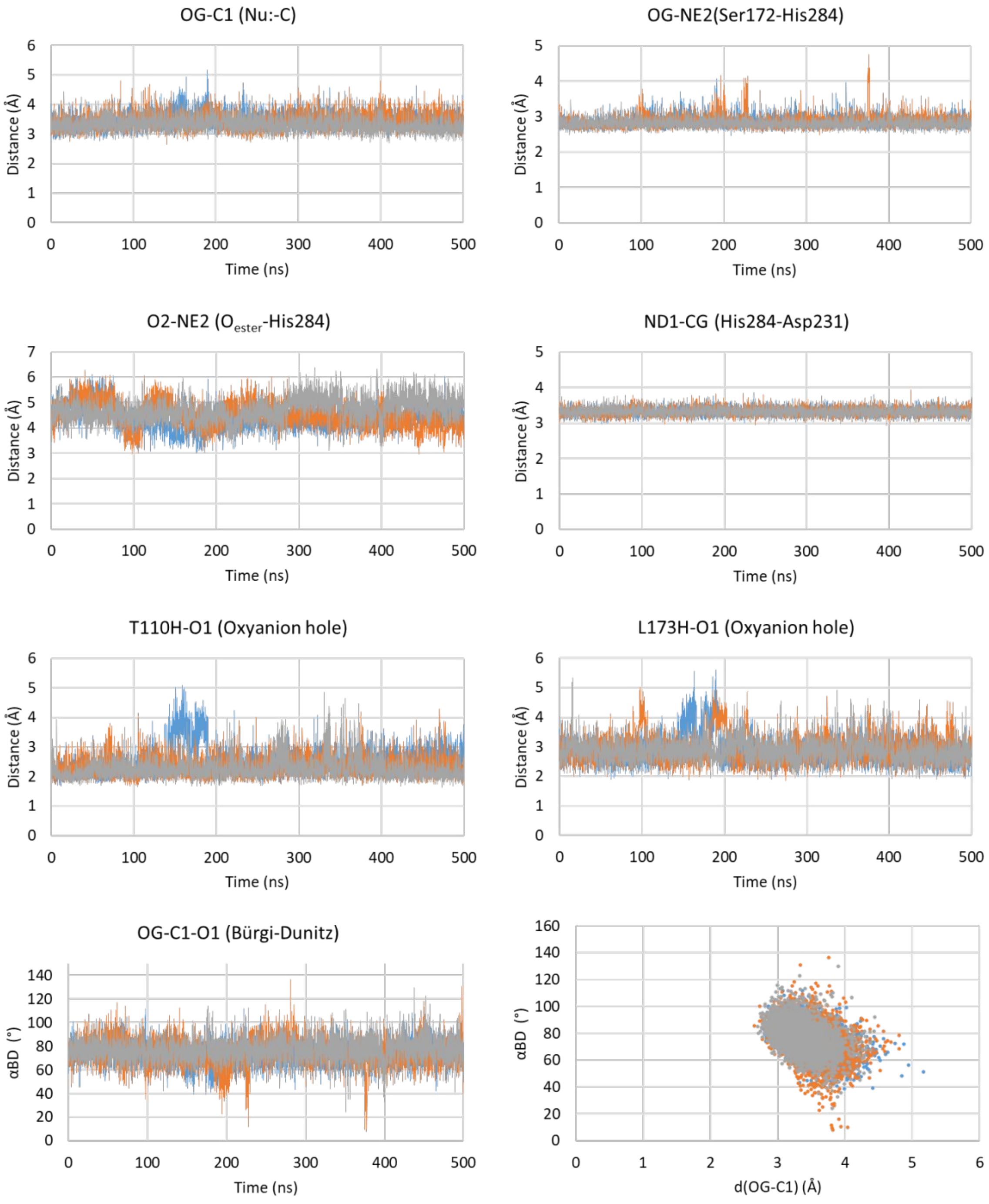

Figure S5. Time-dependent evolution of key distances and angles of the protein:substrate complex along de 500 ns of unbiased MD simulation for the replicas (blue, orange and grey for replicas 1, 2 and 3, respectively) of model pNPB-RCL. Lower right panel: relationship between αBD and the distance of the nucleophilic attack d(OG-C1).

Time-dependent evolution of key distances and angles in the E:S of the pNC-RCL$_A$ model

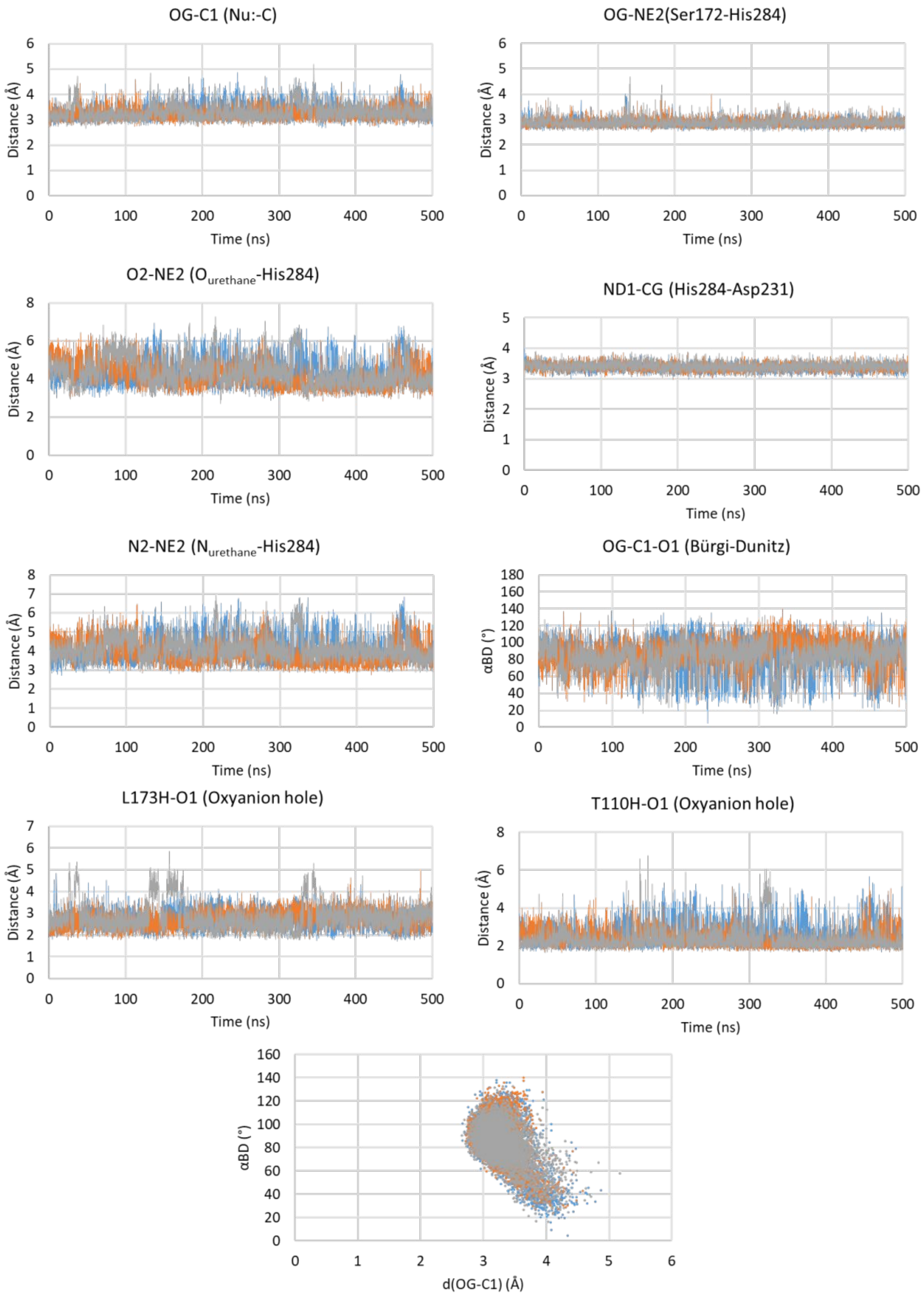

Figure S6. Time-dependent evolution of key distances and angles of the protein:substrate complex along de 500 ns of unbiased MD simulation for the replicas (blue, orange and grey for replicas 1, 2 and 3, respectively) of model pNC-RCL$_A$. Lowermost panel: relationship between αBD and the distance of the nucleophilic attack d(OG-C1).

Time-dependent evolution of key distances and angles in E:S of the pNC-RCL$_B$ model

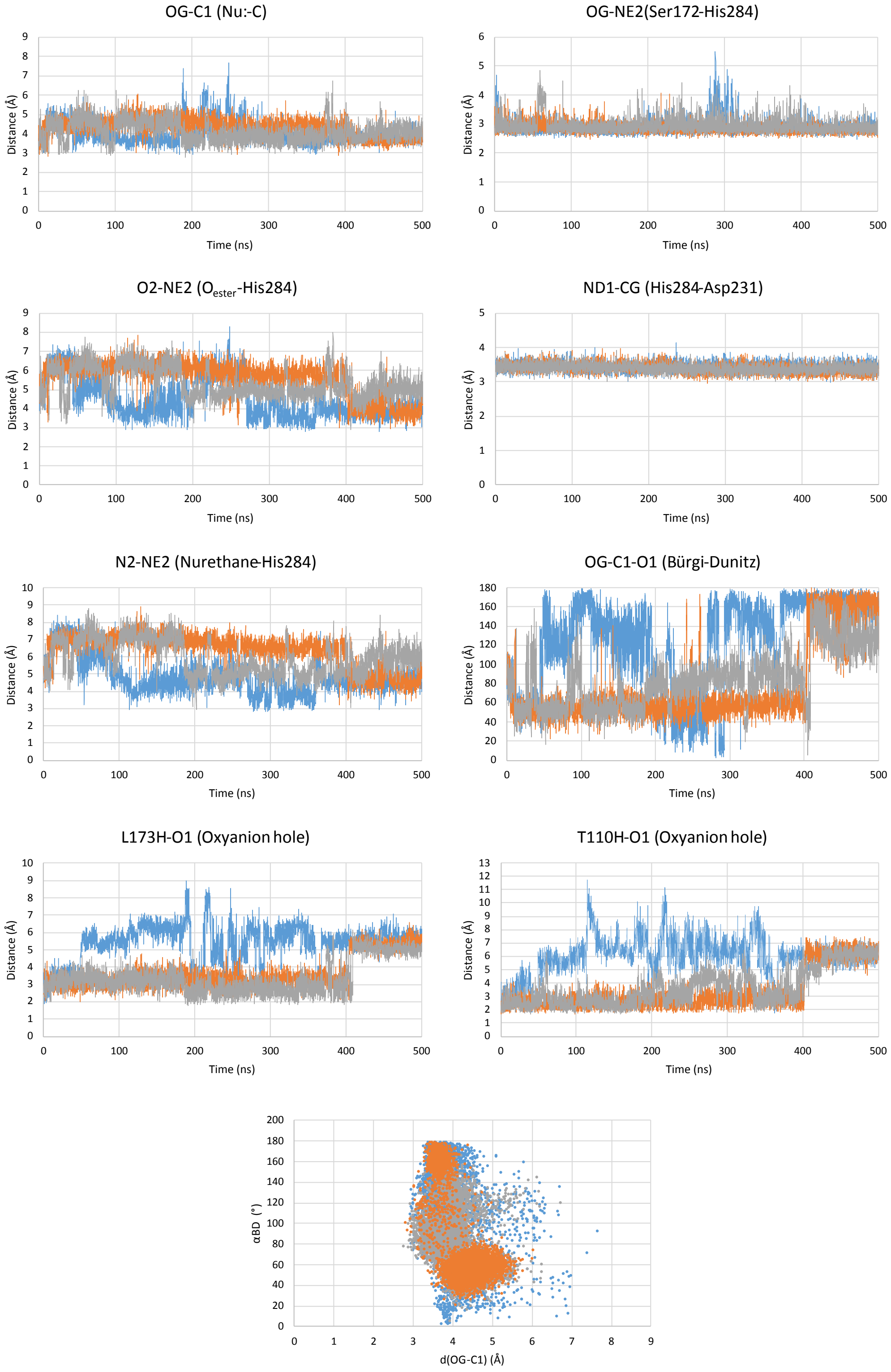

Figure S7. Time-dependent evolution of key distances and angles of the protein:substrate complex along de 500 ns of unbiased MD simulation for the replicas (blue, orange and grey for replicas 1, 2 and 3, respectively) of model pNC-RCL$_A$. Lowermost panel: relationship between αBD and the distance of the nucleophilic attack d(OG-C1).

Population analysis for the Bürgi-Dunitz angle and for the distances established for substrates and oxyanion hole

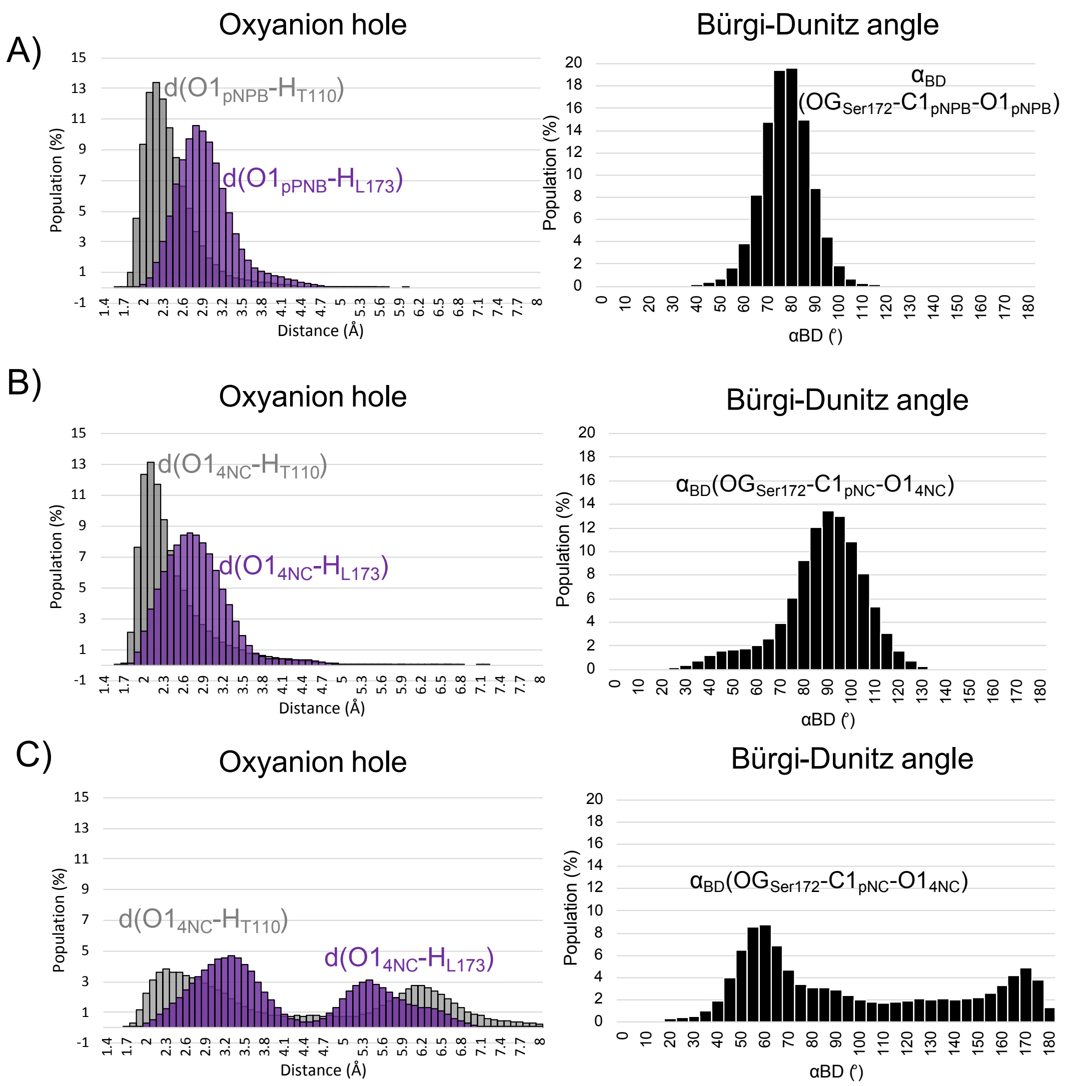

Figure S8. Population analysis for the Bürgi-Dunitz angle (α_{BD}) and for the distances between atom O1 in systems of pNPB-RCL (A), pNC-RCL$_A$ (B) and pNC-RCL$_B$ (C) and oxyanion hole atoms H_{T110} (purple) and H_{L173} (grey).

Time-dependent evolution and population analysis of Phe113 side chain conformers

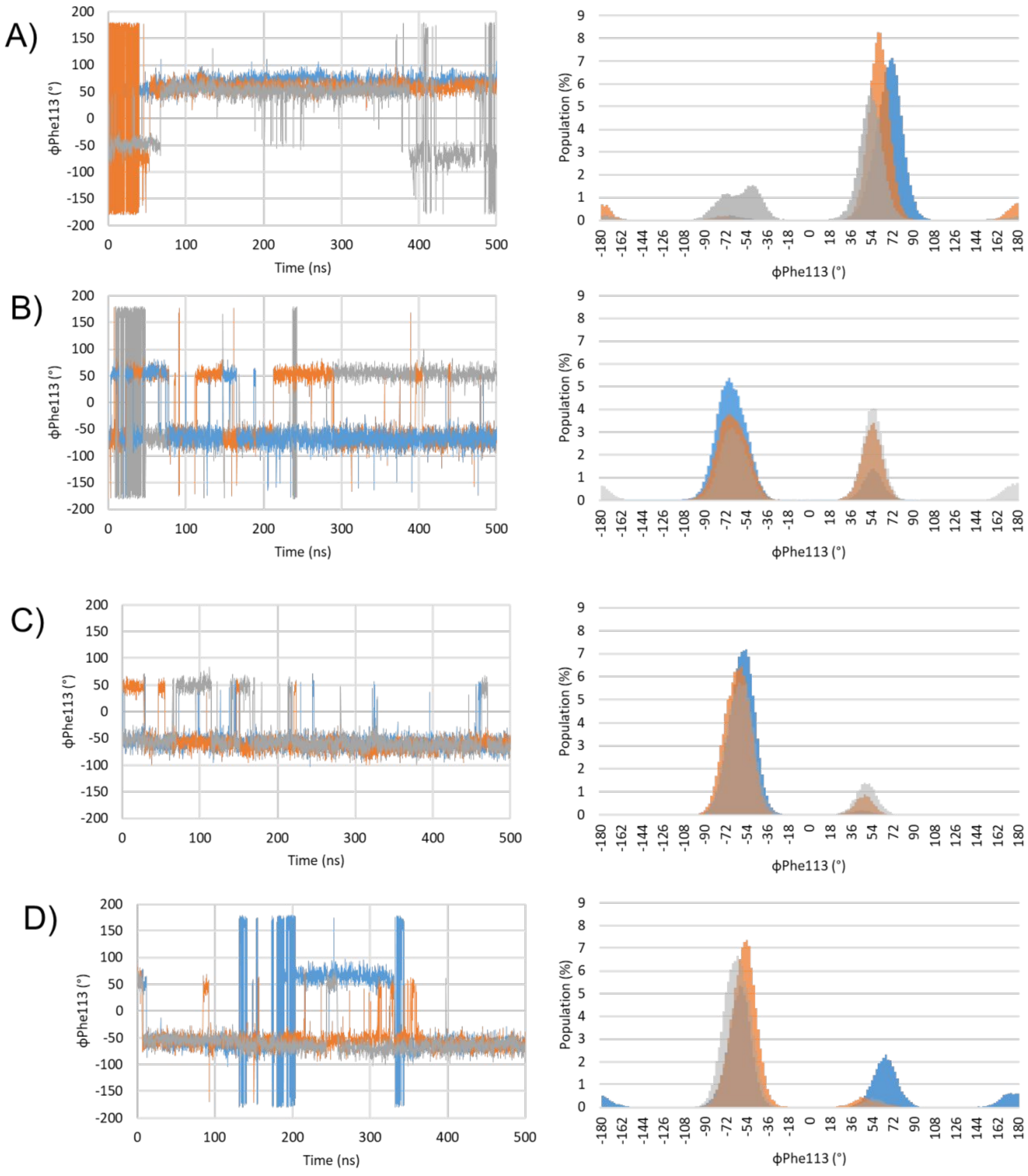

Figure S9. Time-dependent evolution and population analysis of Phe113 side-chain conformers identified based on the φ-dihedral angle (C-Cα-Cβ-Cγ) for the 500 ns of unbiased MD simulations of systems A) Apo enzyme and solvent with high concentration of pNPB, B) pNPB:RCL, C) pNC:RCL$_A$ and D) pNC:RCL$_B$, in blue, orange and grey for replicas 1, 2 and 3, respectively.

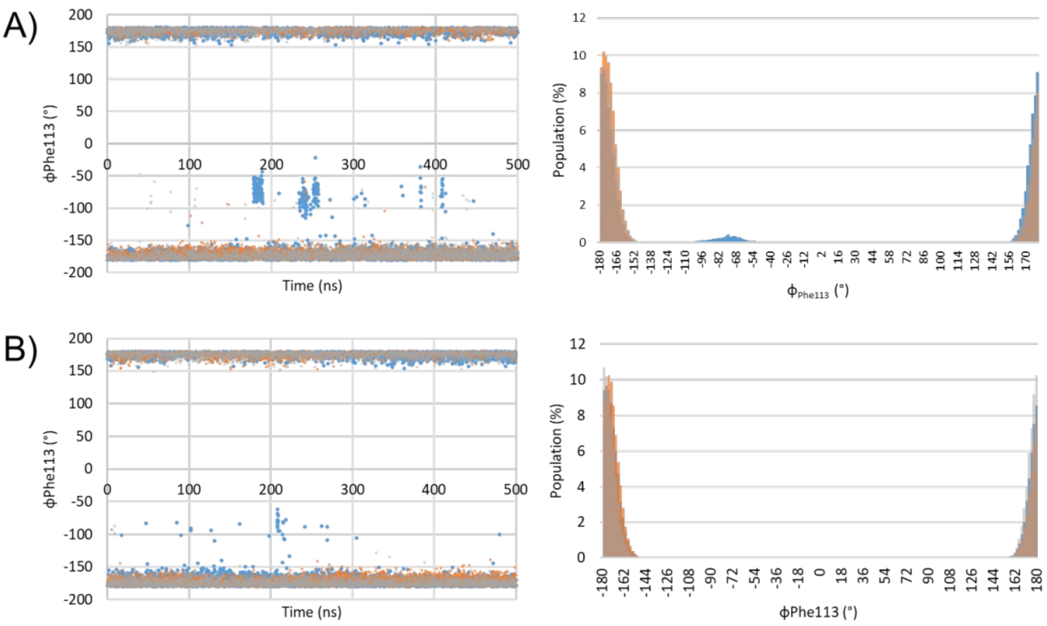

Figure S10. Time-dependent evolution and population analysis of Phe113 side-chain conformers identified based on the φ-dihedral angle (C-Cα-Cβ-Cγ) for the 500 ns of unbiased MD simulations of apoenzyme in the absence of substrate in the solvent (A) and 500 ns of constrained hydrogen bond between Arg114 and Asp11 at high concentration of pNPB substrate (B). Colors blue, orange and grey are for replicas 1, 2 and 3, respectively.

Protein-Substrate interaction energies

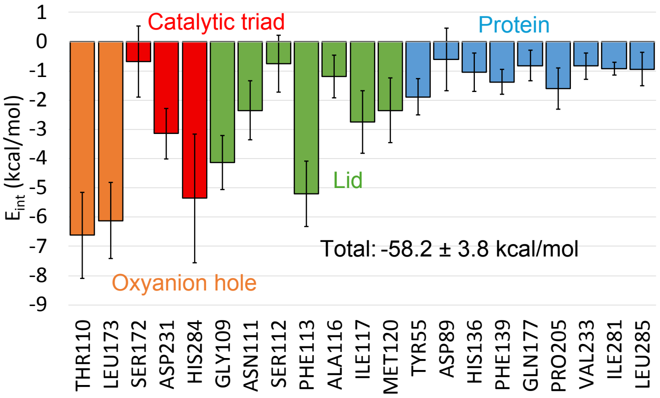

Figure S11. Protein-Substrate interaction energies (E_{int}) decomposed by residue for system pNC-RCL$_A$. Analysis done on 150,000 frames from three independent replicas of 500 ns long simulations.

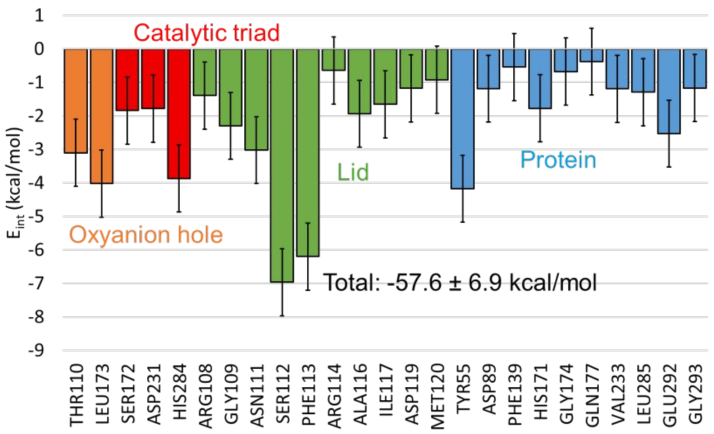

Figure S12. Protein-Substrate interaction energies (E_{int}) decomposed by residue for system pNC-RCL$_B$. Analysis done on 150,000 frames from three independent replicas of 500 ns long simulations.

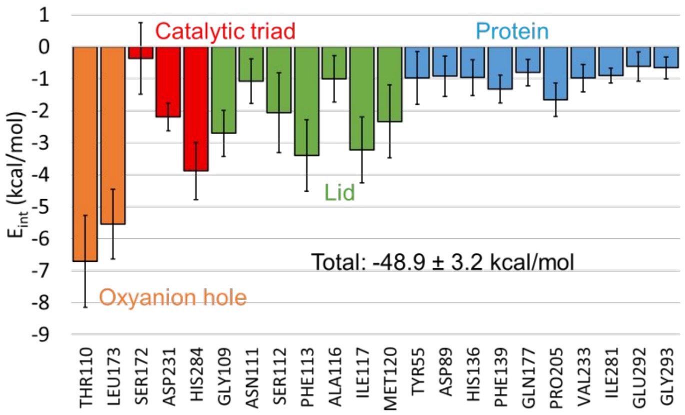

Figure S13. Protein-Substrate interaction energies (E_{int}) decomposed by residue for system pNPB-RCL. Analysis done on 150,000 frames from three independent replicas of 500 ns long simulations.

3. QM/MM simulations

Definition of the QM region in the active site of RCL

The QM region included the side chains of the catalytic triad residues Ser172, His284, and Asp231, as well as all the substrate atoms. Three-link atoms[2] were inserted at the QM/MM boundary where they crossed covalent bonds, between atoms Cα-Cβ for Ser172, His284, and Asp231. In the deacylation step, the leaving group was removed from the model, and four water solvent molecules were placed into the corresponding cavity. One of these molecules was included in the QM region as an active participant in the hydrolysis step.

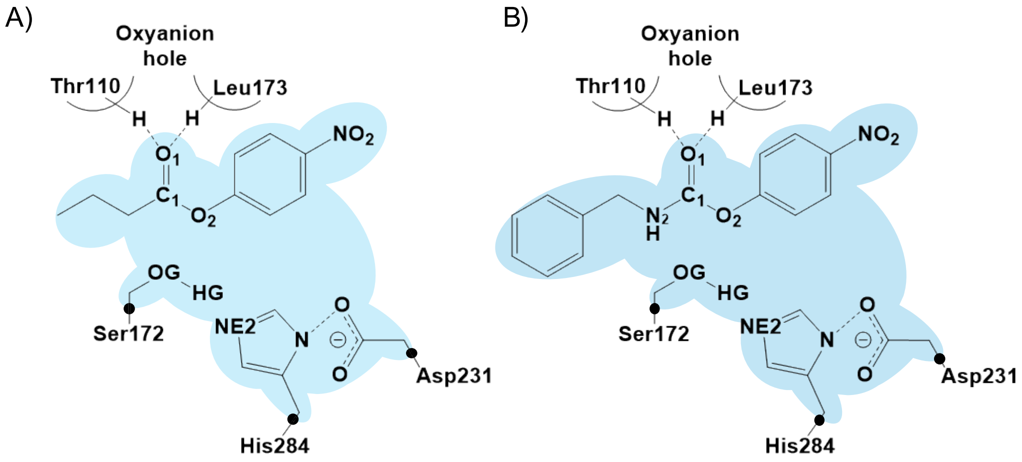

Figure S14. Schematic representation of the active site of RCL in complex with ester benchmark molecule pNPB (A) and model PUR compound pNC (B) in the non-covalent reactant complex, RC. Light blue region contains atoms treated quantum mechanically. Quantum link atoms are represented as black dots.

Detailed information on the exploration of individual potential energy surfaces (PES)

A combination of different distances was controlled during the exploration of all chemical steps explored reaction for all systems under study, that is, four chemical steps for the benchmark pNPB-RCL system, seven for the esterase path of pNC-RCL$_A$, one for the esterase path of pNC-RCL$_B$, and two for the amidase path pNPB-RCL$_A$. The first step for all systems proceeds by the same mechanism, that is, nucleophilic attack of Ser172 to the carbonyl group of the substrate. For this step, all PESs were generated by controlling the antisymmetric combination of the distance between OG and HG of Ser172 and the proton acceptor NE2 of His284, together with the formation of the bond between C1 of the substrate and OG of Ser172, directing the first acylation step. For system pNC-RCL$_B$ this step was the only one performed, while the other systems were explored further. For the amidase pathway of pNC-RCL$_A$ the reaction coordinates used to obtain the PESs of the second step were obtained by controlling the antisymmetric combination of the distances between NE2 of His284, hydrogen atom HG and nitrogen N2 from the substrate, together with the carbon-nitrogen bond (C1-N2) distance from the substrate, characterizing the removal of the benzylamine leaving group. For esterase pathways of systems pNPB-RCL and pNC-RCL$_A$ the PESs for the second to fourth steps were obtained by controlling the same reaction coordinates: for the second step, by controlling the antisymmetric combination of the distances between NE2 of His284, hydrogen atom HG and oxygen OG from Ser172, together with the carbon-oxygen bond (C1-O2) distance from the substrate, characterizing the removal of the *p*-nitrophenol leaving group. In the third step, the PES was generated by scanning the antisymmetric combination of distances between oxygen O_{WAT} from the water molecule, $H1_{WAT}$ from the same water and nitrogen NE2 from His284, together with the distance corresponding to the nucleophilic attack of O_{WAT} to substrate atom C1, comprising the first hydrolysis step. The fourth reaction step was explored by controlling the antisymmetric combination of distances between the nitrogen NE2 of His284, $H1_{WAT}$ hydrogen and atom OG from Ser172, together with elongating the distance between oxygen OG and carbon C1 from the substrate, reforming the catalytic residues' original configuration and finishing the deacetylation of the protein by releasing the hydrolysis products. It is herein proposed that the reaction for system pNC-RCL$_A$ could proceed further to produce decarboxylated final products, requiring three additional chemical steps to do so. The reaction coordinates controlled to produce the PES for the first decarboxylation step were the antisymmetric combination of distances between oxygen O^{WAT}, now bound to the released hydrolysis product, hydrogen $H2_{WAT}$ and nitrogen NE2 from His284. Then, the next PES was done by controlling the antisymmetric combination of distances between nitrogen NE2 from His284, hydrogen $H2_{WAT}$ bound to His284 and substrate atom N2, forming an intermediate with zwitterionic nature. The final step's PES was generated by controlling the elongation of the bond between nitrogen N2 and carbon C1 from the substrate, releasing the decarboxylated benzylamine product and a carbon dioxide (CO_2) molecule. During the exploration of all PES, a 5000 $kJ \cdot mol^{-1} \cdot Å^{-2}$ harmonic constraint was used to maintain the proper interatomic distances along the reaction coordinate, and a series of conjugate gradient and L-BFGS-B optimization algorithms were applied to obtain the final potential energy of the minimized constrained geometry. The distance evolution was controlled by applying a

0.05 Å change for coordinates involving light atoms and a 0.1 Å change when two heavy atoms were involved.

Free energy surfaces computed for all chemical steps calculated for the pNPB-RCL system

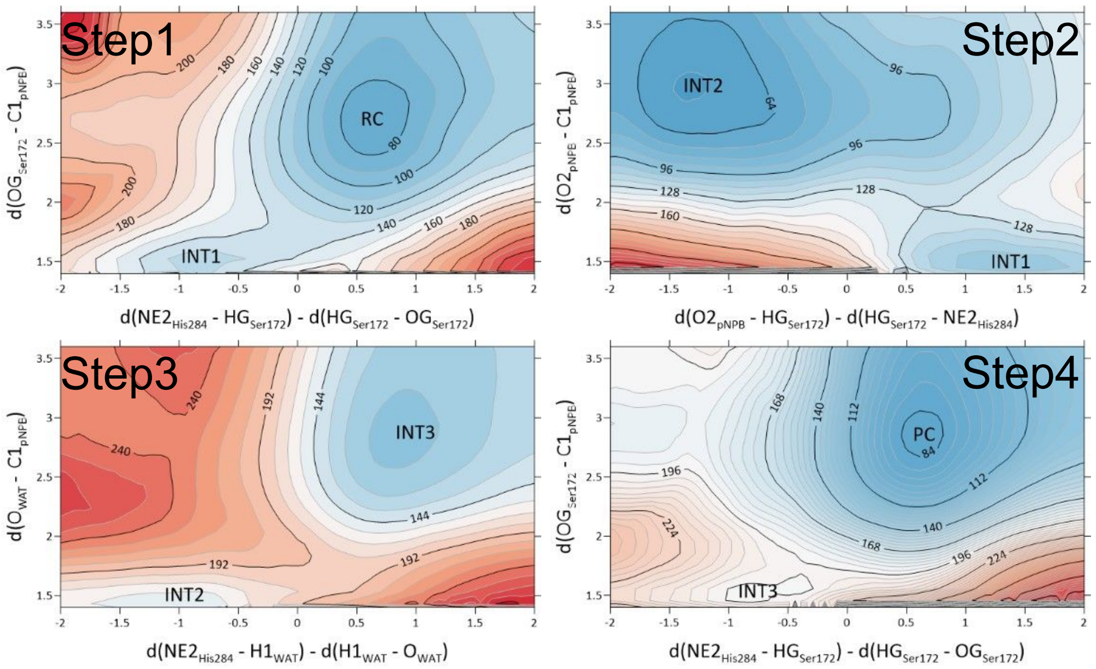

Figure S15. Free energy surfaces computed for all chemical steps calculated for system pNPB-RCL at M06-2X:AM1/MM level.

Free energy surfaces computed for all chemical steps calculated for the pNC-RCL$_A$ system

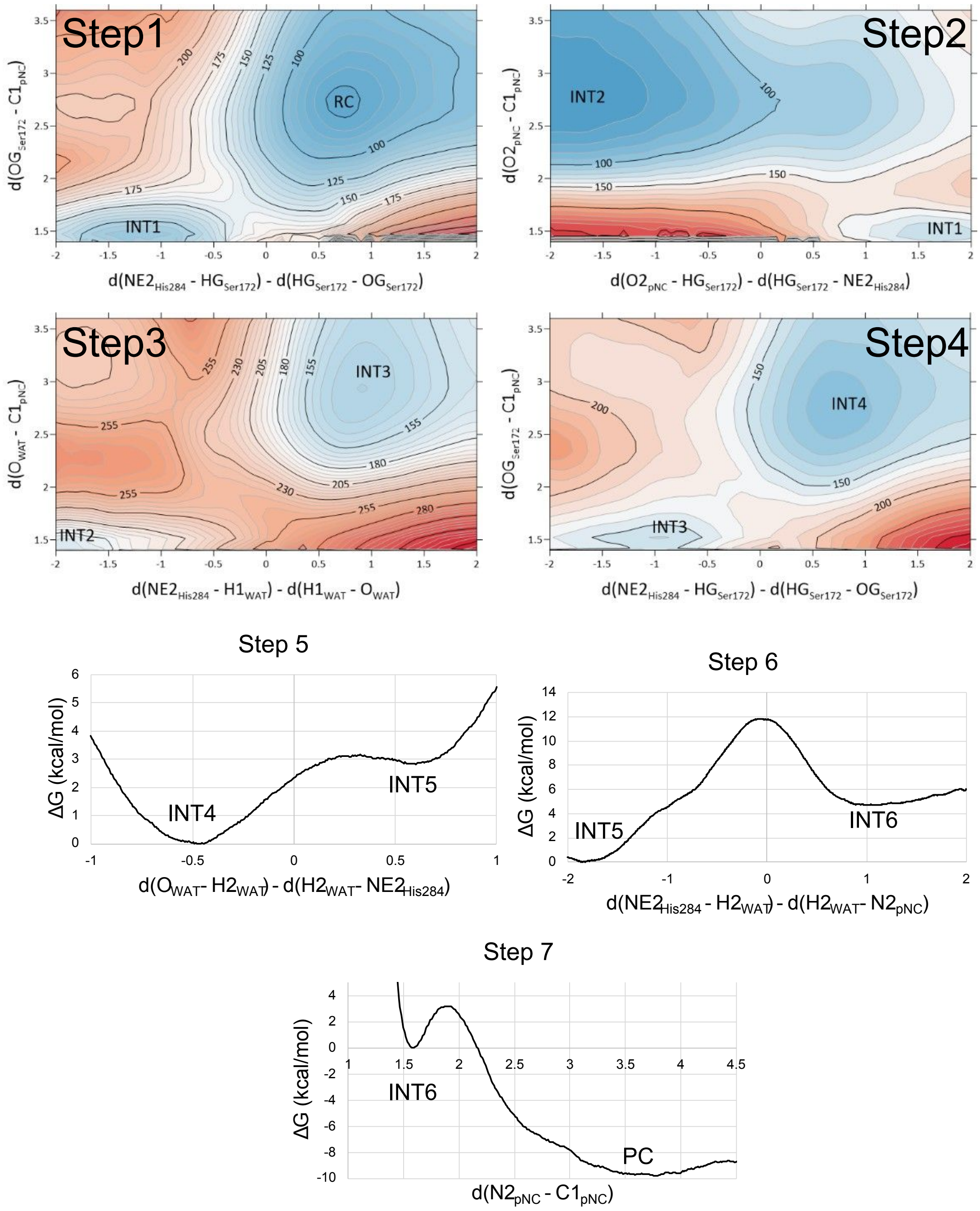

Figure S16. Free energy surfaces computed for all chemical steps calculated for system pNC-RCL$_A$ at M06-2X:AM1/MM level.

Free energy surfaces computed for all chemical steps calculated for the pNC-RCL_B system

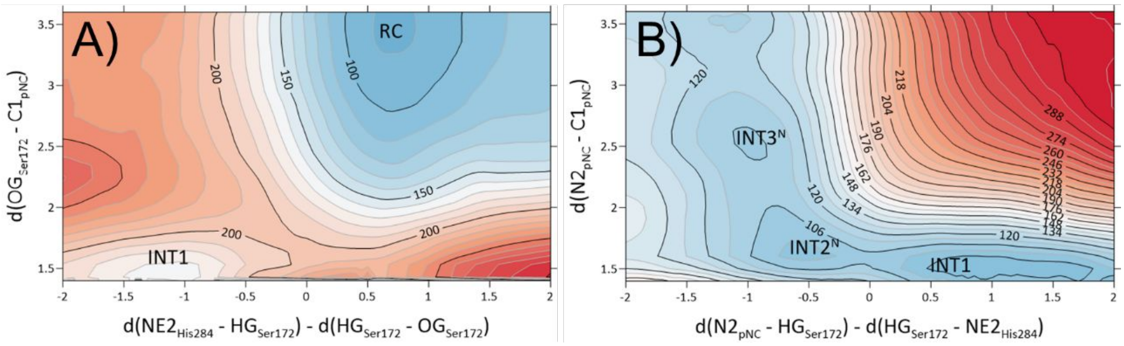

Figure S17. Free energy surfaces computed for the chemical steps of the acylation, calculated for systems **(A)** pNC-RCL_B (step 1) and **(B)** step 2 of the amidase pathway for pNC-RCL_A at M06-2X:AM1/MM level.

PMF window overlap analysis

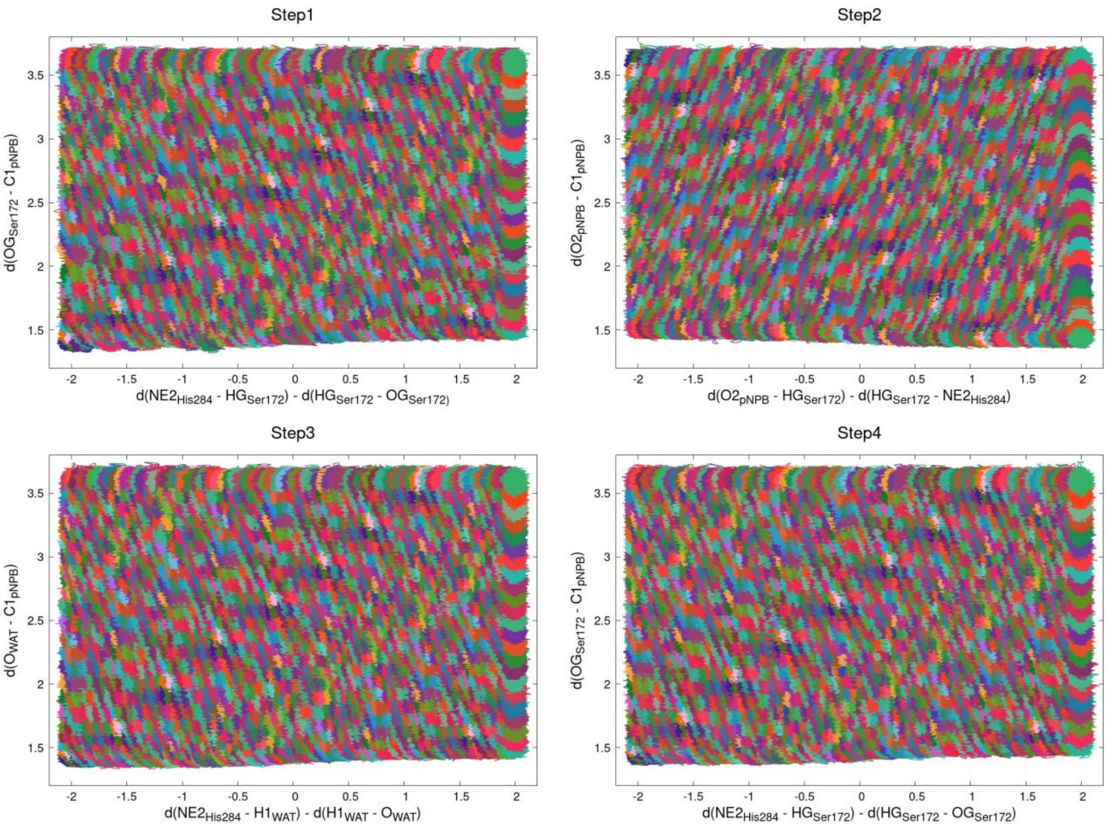

Figure S18. Window overlap analysis for the PMFs computed for the calculation of the free energy surfaces of system pNPB-RCL.

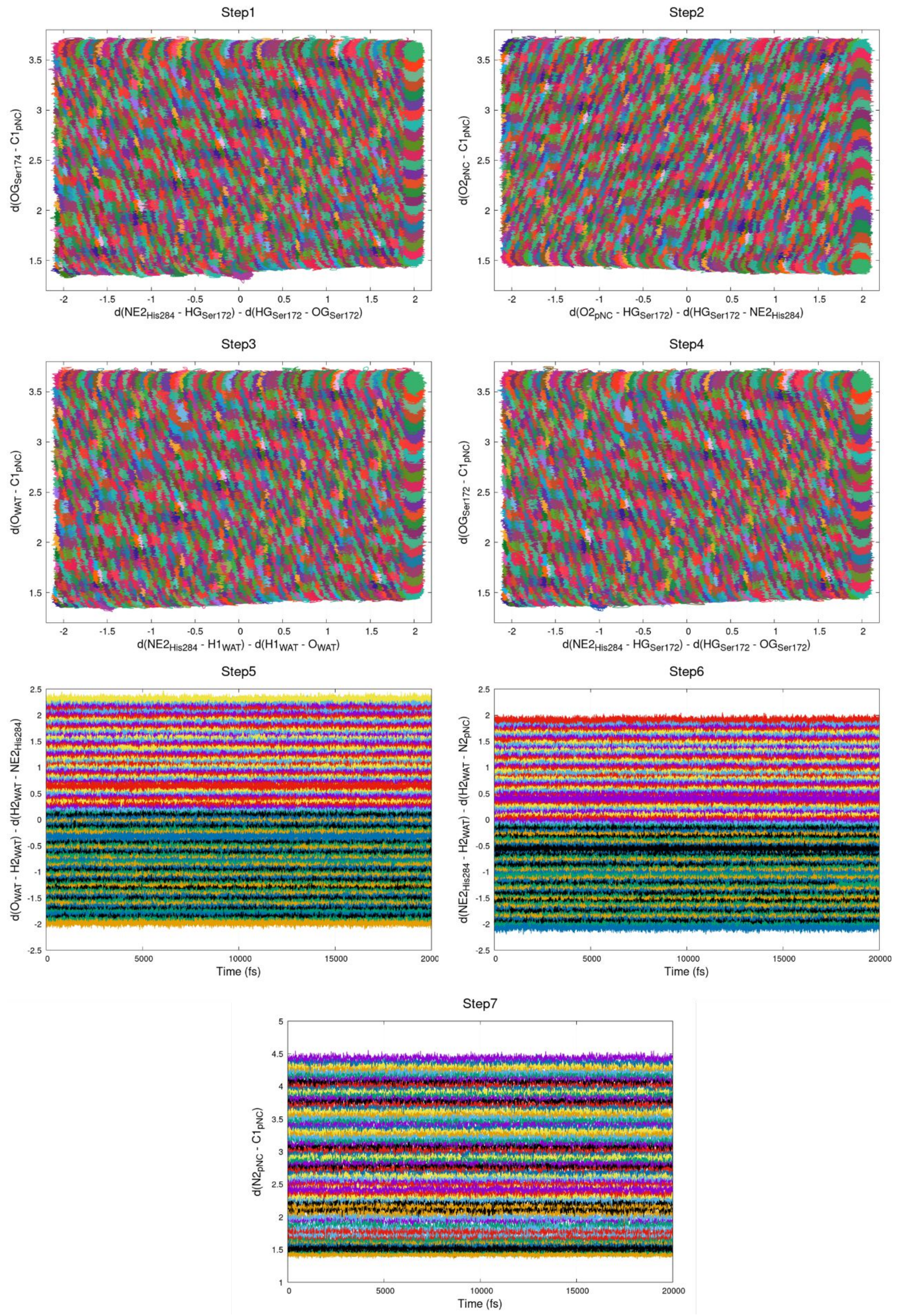

Figure S19. Window overlap analysis for the PMFs computed for the calculation of the free energy surfaces of the esterase pathway of system pNC-RCL$_A$.

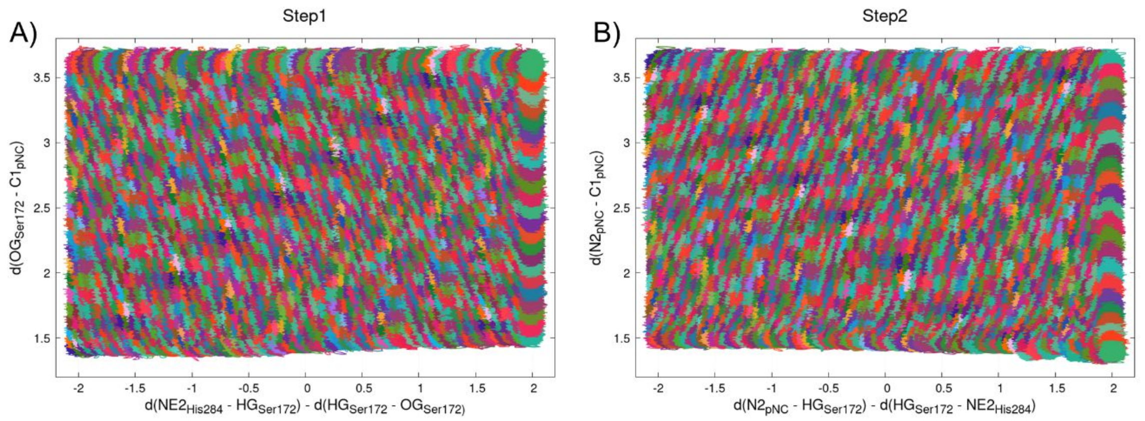

Figure S20. Window overlap analysis for the PMFs computed for the calculation of the free energy surfaces of systems **(A)** pNC-RCL$_B$ (step 1) and **(B)** step 2 of the amidase pathway for pNC-RCL$_A$

Key distances for the structures optimized along the degradation of pNPB

Table S5. Key distances (in Å) for the structures optimized along the degradation of pNPB catalyzed by RCL, computed at M06-2X(6-31+G(d,p))/AMBER level.

	Acylation					
	Step1			Step2		
	RC	TS1	INT1	INT1	TS2	INT2
S172:OG-pNPB:C1	2.60	1.71	1.55	1.48	1.4	1.37
S172:OG-S172:HG	0.99	1.38	1.68	2.09	2.39	2.69
S172:HG-H284:NE2	1.73	1.18	1.06	1.03	1.05	1.6
pNPB:O1-T110:H	1.91	2.14	2.13	2.03	2.01	1.87
pNPB:O1-L173:H	2.46	2.65	2.13	2.01	2.01	2.13
pNPB:O2-pNPB:C1	1.36	1.45	1.5	1.55	2.1	2.56
pNPB:O2-S172:HG	2.84	2.66	2.60	2.23	1.84	1.04

	Hydrolysis					
	Step3			Step4		
	INT2	TS3	INT3	INT3	TS4	INT4
S172:OG-pNC:C1	1.36	1.45	1.53	1.61	1.76	2.74
pNPB:O1-T110:H	1.91	1.98	2.03	2.00	1.98	1.96
pNPB:O1-L173:H	2.71	2.59	2.49	2.50	2.53	2.57
WAT:O-pNPB:C1	2.82	1.72	1.46	1.43	1.40	1.33
WAT:O-WAT:H1	0.98	1.13	1.79	2.22	2.39	2.84
WAT:H1-H284:NE2	1.78	1.38	1.04	1.04	1.08	1.6
WAT:H1-S172:OG	2.88	2.38	2.18	1.74	1.56	1.01

Key distances for the structures optimized along the degradation of pNC_A via esterase activity

Table S6. Key distances (in Å) for the structures optimized along the degradation of pNC in Pose A catalyzed by RCL according to the esterase pathway, computed at M06-2X(6-31+G(d,p))/AMBER level.

**Pose A**	Acylation					
	Step1			Step2		
	RC	TS1	INT1	INT1	TS2	INT2
S172:OG-pNC:C1	2.56	1.76	1.48	1.47	1.43	1.37
S172:OG-S172:HG	0.98	1.23	1.93	1.95	2.07	2.68
S172:HG-H284:NE2	1.83	1.29	1.03	1.03	1.03	2.68
pNC:O1-T110:H	1.88	2.23	2.14	2.34	2.29	2.03
pNC:O11-L173:H	2.46	2.08	2.11	2.31	2.32	2.62
pNC:O2-pNC:C1	1.37	1.42	1.49	1.58	1.85	2.71
pNC:O2-S172:HG	2.76	2.74	2.80	2.63	2.49	0.98

**Pose A**	Hydrolysis					
	Step3			Step4		
	INT2	TS3	INT3	INT3	TS4	INT4
S172:OG-pNC:C1	1.35	1.41	1.48	1.51	1.71	2.71
pNC:O1-T110:H	1.91	2.05	2.06	2.36	2.36	2.11
pNC:O1-L173:H	2.87	3.00	2.45	2.22	2.26	3.19
WAT:O-pNC:C1	2.68	1.73	1.46	1.44	1.40	1.34
WAT:O-WAT:H1	0.99	1.37	2.13	2.40	2.46	2.35
WAT:H1-H284:NE2	1.76	1.15	1.03	1.06	1.27	2.0
WAT:H1-S172:OG	2.72	2.82	2.03	1.67	1.25	0.98

**Pose A**	Decarboxylation								
	Step5			Step6			Step7		
	INT4	TS5	INT5	INT5	TS5	INT6	INT6	TS7	PC
WAT:O-pNC:C	1.34	1.33	1.3	1.30	1.24	1.22	1.21	1.18	1.17
WAT:H2-WAT:O	1.11	1.17	1.49	1.75	2.61	2.64	2.76	2.08	3.41
WAT:H2-H284:NE2	1.41	1.33	1.1	1.05	1.37	1.95	2.49	2.47	2.39
WAT:H2-pNC:N2	2.55	2.59	2.78	2.74	1.29	1.05	1.03	1.02	1.02
pNC:N2-pNC:C8	1.36	1.37	1.39	1.39	1.53	1.66	1.74	2.17	2.5

Key distances for the structures optimized along the degradation of pNC_A via amidase activity

Table S7. Key distances (in Å) for the structures optimized along second acylation step for amidase activity of pNC-RCL_A, computed at M06-2X(6-31+G(d,p))/AMBER level.

**Pose A**	Acylation					
	Step2					
	INT1	TS2N	INT2N	INT2N	TS3N	INT3N
S172:OG-pNC:C1	1.47	1.57	1.41	1.41	1.34	1.33
S172:OG-S172:HG	2.42	2.53	2.44	2.42	2.72	2.82
S172:HG-H284:NE2	1.05	1.25	1.78	1.95	2.16	2.21
pNC:O1-T110:H	1.95	1.99	1.90	1.89	1.91	1.93
pNC:O1-L173:H	2.84	2.89	2.86	2.85	2.70	2.60
pNC:N2-pNC:C8	1.52	1.57	1.65	1.67	2.26	2.51
pNC:N2-S172:HG	2.00	1.41	1.07	1.05	1.03	1.02

Key distances for the structures optimized along the degradation of pNC_B

Table S8. Key distances (in Å) for the structures optimized along the first acylation step for system pNC-RCL_B, computed at M06-2X(6-31+G(d,p))/AMBER level.

**Pose B**	Acylation		
	Step1		
	RC	TS1	INT1
S172:OG-pNC:C1	2.60	1.73	1.48
S172:OG-S172:HG	1.00	1.22	1.67
S172:HG-H284:NE2	1.72	1.28	1.05
pNC:O1-T110:H	3.64	3.99	3.98
pNC:O1-L173:H	2.26	1.96	1.93

CHarges from ELectrostatic Potentials using a Grid-based method for optimized structures

Table S9. CHarges from ELectrostatic Potentials using a Grid-based method (CHelpG) provided in atomic units, computed for key atoms for optimized structures at the M06-2X(6-31+G(d,p))AMBER level along the first acylation step for system pNC-RCL_B.

		Atom					
		$C1_{Sub}$	$O2_{Sub}$	$O1_{Sub}$	$N2_{Sub}$	OG_{Ser172}	$NE2_{His284}$
pNC	RC	0.865	-0.371	-0.710	-0.422	-0.658	-0.162
	TS1	1.078	-0.513	-0.843	-0.675	-0.656	-0.103
	I1	1.208	-0.563	-0.914	-0.851	-0.673	-0.091

Interaction energy calculated for INT2(esterase) and INT3^N in system pNC-RCL$_A$

Table S10. Total interaction energy (in kcal/mol) calculated for INT2(esterase) and INT3^N in system pNC-RCL$_A$. Analysis done over 200 structures of 20 ps AM1/AMBER NVT MD simulation.

Regions	INT2(esterase)	INT3^N
1-36	-0.3 ± 0.5	1.1 ± 1.2
α-helix(37-54)	-2.4 ± 0.3	1.2 ± 0.3
55-66	-0.2 ± 0.8	-1.9 ± 0.8
α-helix(67-74)	0.5 ± 0.2	-0.3 ± 0.1
75-77	0.0 ± 0.0	0.0 ± 0.0
β-strand(78-85)	0.4 ± 0.3	0.1 ± 0.2
α-helix(86-89)	-5.0 ± 1.1	0.6 ± 2.2
β-strand(90-97)	0.7 ± 0.2	-0.6 ± 0.2
98-101	0.0 ± 0.0	0.0 ± 0.0
β-strand(102-108)	7.6 ± 1.0	-3.5 ± 1.5
109-111	-4.4 ± 1.7	-5.9 ± 3.2
α-helix(112-119)	-4.0 ± 1.7	-15.5 ± 2.4
120-123	-3.8 ± 0.6	-2.1 ± 0.7
β-strand(124-126)	0.0 ± 0.1	0.1 ± 0.1
127-133	0.0 ± 0.0	0.0 ± 0.0
β-strand(134-136)	-1.3 ± 0.6	-0.7 ± 0.2
α-helix(137-161)	-5.5 ± 0.5	-2.2 ± 0.5
162-164	0.0 ± 0.0	0.0 ± 0.0
β-strand(165-171)	-4.4 ± 1.8	-1.2 ± 1.3
α-helix(172-189)	1.3 ± 1.4	3.6 ± 1.7
190-196	0.0 ± 0.0	0.0 ± 0.0
β-strand(197-202)	-1.1 ± 0.5	-2.9 ± 0.5
203-208	-2.0 ± 0.4	1.5 ± 0.4
α-helix(209-219)	0.0 ± 0.0	0.0 ± 0.0
220-222	0.1 ± 0.1	0.3 ± 0.0
β-strand(223-228)	1.1 ± 0.4	0.9 ± 0.5
229-231	-0.4 ± 1.2	-3.6 ± 1.6
α-helix(232-236)	-2.3 ± 0.6	-1.2 ± 0.5
237	0.3 ± 0.1	-0.1 ± 0.1
α-helix(238-242)	0.0 ± 0.1	-0.1 ± 0.1
243-247	-0.1 ± 0.1	0.8 ± 0.2
β-strand(248-254)	0.0 ± 0.0	0.0 ± 0.0
255-257	0.0 ± 0.0	0.0 ± 0.0
β-strand(258-262)	0.0 ± 0.0	0.0 ± 0.0
263-270	-0.1 ± 0.1	-0.3 ± 0.2
α-helix(271-276)	-0.1 ± 0.1	-0.2 ± 0.0
277-280	-1.5 ± 0.3	0.8 ± 0.7
α-helix(281-284)	-9.4 ± 2.3	-1.9 ± 2.4
285-305	-0.7 ± 0.8	-3.9 ± 1.5
**SUM**	-37.1 ± 4.9	-37.4 ± 6.6

Potential energy calculated for INT2(esterase) and $INT3^N$ of $pNC\text{-}RCL_A$ in vacuum

Table S11. Potential energy calculated for INT2(esterase) and $INT3^N$ QM structures based on $pNC\text{-}RCL_A$ system at M06-2X(6-31+G(d,p)) level in vacuum.

	E_{elec} (Hartree)	ΔE_{elec} (kcal/mol)
RC	-1560.290425	0.0
INT2 (esterase)	-1560.289533	0.6
$INT3^N$	-1560.262295	17.7

Free energy derived from the FELs.

Table S12. Free energies (in kcal/mol) derived from the FELs for all systems discussed in the present work. Values of reported free energies include ZPE corrections.

pNPB-RCL	
RC	0.0
TS1	12.9
INT1	11.3
TS2	18.6
INT2	1.8
INT2'	0.0
TS3	19.3
INT3	11.4
TS4	10.8
PC	-15.2

$pNC\text{-}RCL_A$ (amidase)	
RC	0
TS1	17.2
INT1	9.2
$TS2^N$	11.2
$INT2^N$	12.6
$TS3^N$	14.4
$INT3^N$	13.4

$pNC\text{-}RCL_A$ (esterase)	
RC	0.0
TS1	17.2
INT1	9.2
TS2	15.6
INT2	-8.5
INT2'	0.0
TS3	16.3
INT3	-7.2
TS4	-2.2
INT4	-15.5
INT4	0.0
TS5	2.0
INT5	3.4
TS6	13.6
INT6	8.0
TS7	10.3
PC	-2.9

$pNC\text{-}RCL_B$	
RC	0
TS1	23.7
INT1	17.9

Electrostatic potential (V_{elec})

Table S13. Average electrostatic potential, V_{elec} (in kJ·mol^{-1}·e^{-1}), computed on the key atoms of the active site of RCL for 200 structures generated during 20 ps of MD simulations. V_{elec} is computed for all amino acids of the protein except three that belong to the catalytic triad and are part of the QM subset of atoms.

pNPB

V_{elec}	C1	std	O2	std	O1	std	OG	std	NE2	std
RC	430.46	19.02	398.06	15.71	493.99	21.15	474.98	17.04	461.94	17.01
TS1	469.88	18.61	413.56	15.38	543.15	22.18	475.78	17.97	448.55	14.9
INT1	500.12	17.52	434.36	15.41	580.91	20.79	492.38	14.27	449.03	15.31

V_{elec}	C1	std	Owat	std	O1	std	OG	std	NE2	std
INT2	353.00	26.25	308.73	22.29	396.49	35.83	350.22	23.76	339.69	25.81
TS3	361.84	20.47	325.71	20.05	388.64	23.82	364.98	20.14	347.21	22.05
INT3	337.51	20.59	288.79	16.89	359.19	22.19	352.95	21.72	346.19	22.4

pNC in pose A

V_{elec}	C1	std	O2	std	O1	std	OG	std	NE2	std
RC	438.8	18.12	413.63	14.21	481.01	21.07	466.26	17.86	474.19	18.28
TS1	503.4	15.6	463.34	13.5	548.21	20.39	512.96	15.55	484.62	16.14
INT1	493.87	13.64	447.46	12.77	548.17	15.9	489.51	15.14	478.2	17.13

V_{elec}	C1	std	Owat	std	O1	std	OG	std	NE2	std
INT2	379.01	16.9	337.82	13.83	440.27	19.61	376.07	16.69	370.86	17.97
TS3	404.25	17.93	360.32	14.1	421.94	19	402.67	18.79	381.64	16.84
INT3	379.01	16.9	337.82	13.83	440.27	19.61	376.07	16.69	370.86	17.97

4. Cartesian coordinates of QM atoms for the optimized transition states.

Table S14. Cartesian coordinates (in Å) of QM atoms for the transition states corresponding to the acylation steps of system pNPB-RCL, optimized at M06-2X(6-31+G(d,p))/MM level of theory.

TS1 (vi = 498.1i cm^{-1})				TS2 (vi = 103.3i cm^{-1})			
Atom	X	Y	Z	Atom	X	Y	Z
C	34.847	42.479	45.803	C	35.044	43.125	46.006
H	34.748	43.494	45.400	H	34.906	44.069	45.468
H	35.659	42.502	46.533	H	35.888	43.254	46.684
O	33.640	42.123	46.468	O	33.889	42.781	46.764
H	33.828	41.327	47.581	H	33.154	41.414	48.579
C	35.892	34.382	50.928	C	36.135	34.780	51.290
H	36.242	33.648	50.195	H	36.667	34.107	50.611
H	36.783	34.741	51.453	H	36.887	35.265	51.919
C	35.302	35.579	50.171	C	35.425	35.870	50.460
O	34.106	35.916	50.325	O	34.204	36.057	50.584
O	36.093	36.233	49.408	O	36.141	36.618	49.697
C	34.724	38.651	51.561	C	34.323	38.564	52.066
H	34.229	39.297	52.294	H	33.756	39.159	52.784
H	34.191	37.694	51.546	H	33.845	37.586	51.981
C	34.561	39.277	50.195	C	34.206	39.274	50.742
N	34.908	38.596	49.049	N	34.785	38.858	49.560
H	35.320	37.615	49.057	H	35.350	37.940	49.477
C	34.621	39.361	47.993	C	34.444	39.702	48.585
H	34.746	39.071	46.963	H	34.712	39.600	47.543
N	34.105	40.518	48.390	N	33.661	40.656	49.093
C	34.056	40.484	49.772	C	33.498	40.407	50.437
H	33.661	41.307	50.347	H	32.889	41.045	51.058
C	29.984	42.712	45.684	C	30.005	43.306	46.861
H	30.255	43.694	45.322	H	30.270	44.353	46.931
C	28.999	41.956	45.060	C	28.949	42.900	46.074
H	28.455	42.317	44.196	H	28.388	43.615	45.486
C	28.680	40.704	45.573	C	28.589	41.548	46.031
N	27.737	39.857	44.853	N	27.506	41.136	45.183
O	27.335	38.851	45.423	O	27.164	39.961	45.182
O	27.433	40.175	43.718	O	26.973	41.997	44.488
C	29.265	40.204	46.730	C	29.265	40.597	46.797
H	28.962	39.236	47.107	H	28.942	39.562	46.773
C	30.248	40.957	47.345	C	30.342	41.003	47.553
H	30.736	40.621	48.253	H	30.873	40.300	48.176
C	30.651	42.182	46.798	C	30.793	42.351	47.566
O	31.631	42.821	47.475	O	31.880	42.684	48.195
C	32.712	43.519	46.813	C	33.097	43.908	47.001
O	32.506	44.121	45.736	O	32.466	44.351	46.052
C	33.414	44.197	47.990	C	33.505	44.772	48.170
H	32.637	44.833	48.426	H	34.214	45.503	47.747

H	33.667	43.436	48.738		H	32.618	45.329	48.478
C	34.615	45.084	47.694		C	34.156	44.069	49.356
H	35.451	44.517	47.280		H	33.389	43.492	49.881
H	34.323	45.829	46.949		H	34.896	43.350	48.983
C	35.082	45.779	48.973		C	34.820	45.064	50.305
H	35.325	45.042	49.748		H	35.271	44.561	51.164
H	34.301	46.433	49.376		H	34.097	45.787	50.695
H	35.975	46.379	48.791		H	35.608	45.628	49.791
H	35.663	38.474	51.858		H	35.241	38.417	52.435
H	35.291	33.930	51.587		H	35.504	34.257	51.863
H	35.141	41.869	45.067		H	35.251	42.395	45.354

Table S15. Cartesian coordinates (in Å) of QM atoms for the transition states corresponding to the hydrolysis steps of system pNPB-RCL, optimized at M06-2X(6-31+G(d,p))/MM level of theory.

TS3 (vi = 256.5i cm^{-1})					TS4 (vi = 126.8i cm^{-1})			
Atom	X	Y	Z		Atom	X	Y	Z
C	35.006	42.595	45.744		C	34.864	42.478	45.834
H	35.007	43.580	45.260		H	34.699	43.461	45.365
H	35.788	42.598	46.508		H	35.735	42.601	46.491
O	33.748	42.352	46.349		O	33.739	42.150	46.626
H	33.020	41.806	48.544		H	33.887	41.335	47.953
C	35.821	34.550	51.284		C	35.797	34.517	51.281
H	36.260	33.880	50.539		H	36.221	33.847	50.529
H	36.648	34.952	51.876		H	36.632	34.902	51.874
C	35.168	35.738	50.537		C	35.158	35.713	50.564
O	33.973	36.045	50.742		O	33.992	36.077	50.817
O	35.903	36.413	49.745		O	35.889	36.349	49.726
C	34.447	38.619	52.045		C	34.454	38.753	51.918
H	33.935	39.228	52.800		H	33.959	39.447	52.606
H	33.950	37.648	51.994		H	33.894	37.815	51.914
C	34.266	39.320	50.731		C	34.380	39.349	50.529
N	34.700	38.830	49.522		N	34.852	38.677	49.422
H	35.146	37.885	49.420		H	35.260	37.668	49.454
C	34.365	39.714	48.564		C	34.678	39.442	48.347
H	34.560	39.561	47.511		H	34.919	39.172	47.332
N	33.734	40.755	49.092		N	34.108	40.587	48.705
C	33.667	40.519	50.446		C	33.905	40.554	50.068
H	33.200	41.222	51.120		H	33.439	41.378	50.585
C	33.195	43.542	46.969		C	32.965	43.612	47.230
O	32.330	44.124	46.276		O	32.370	44.131	46.244
C	34.215	44.306	47.802		C	34.096	44.330	47.953
H	34.661	43.584	48.494		H	34.494	43.635	48.704
H	35.015	44.670	47.149		H	34.901	44.573	47.256
C	33.635	45.486	48.571		C	33.615	45.607	48.645
H	33.357	46.271	47.864		H	33.418	46.371	47.889
H	32.720	45.179	49.081		H	32.669	45.408	49.155
C	34.657	46.036	49.566		C	34.652	46.131	49.637

H	35.588	46.312	49.060
H	34.895	45.293	50.336
H	34.281	46.928	50.074
O	32.406	42.698	48.237
H	31.578	42.406	47.823
H	35.387	38.467	52.349
H	35.209	34.032	51.882
H	35.247	41.901	45.066

H	35.613	46.311	49.145
H	34.813	45.415	50.451
H	34.331	47.075	50.088
O	32.177	42.986	48.206
H	31.334	42.800	47.769
H	35.373	38.556	52.257
H	35.177	34.010	51.879
H	35.131	41.830	45.121

Table S16. Cartesian coordinates (in Å) of QM atoms for the transition states corresponding to the acylation steps of the esterase pathway of system pNC-RCL$_A$, optimized at M06-2X(6-31+G(d,p))/MM level of theory.

TS1 (vi = 862.2i cm^{-1})			
Atom	X	Y	Z
C	37.715	38.700	26.668
H	38.763	38.558	26.380
H	37.606	39.747	26.987
O	36.880	38.481	25.543
H	36.053	39.394	25.480
C	29.037	41.852	28.281
H	28.734	41.322	29.188
H	29.258	42.885	28.567
C	30.334	41.230	27.747
O	30.398	40.829	26.567
O	31.344	41.193	28.540
C	32.387	42.782	25.340
H	32.647	43.248	24.384
H	31.429	42.263	25.220
C	33.429	41.736	25.659
N	33.378	40.973	26.803
H	32.599	41.006	27.529
C	34.399	40.111	26.778
H	34.582	39.356	27.522
N	35.127	40.263	25.680
C	34.530	41.284	24.964
H	34.917	41.607	24.010
C	35.534	35.979	22.882
H	34.689	36.459	23.361
C	35.432	34.700	22.354
H	34.511	34.135	22.414
C	36.550	34.136	21.748
N	36.444	32.784	21.196
O	35.384	32.200	21.323
O	37.424	32.315	20.634
C	37.766	34.808	21.668
H	38.623	34.327	21.213

TS2 (vi = 143.1i cm^{-1})			
Atom	X	Y	Z
C	37.972	38.587	26.898
H	39.052	38.453	26.779
H	37.811	39.631	27.186
O	37.315	38.351	25.656
H	35.706	39.584	25.218
C	29.138	42.001	28.234
H	28.864	41.531	29.183
H	29.391	43.041	28.460
C	30.397	41.310	27.696
O	30.435	40.859	26.542
O	31.439	41.271	28.468
C	32.318	42.788	25.313
H	32.584	43.251	24.359
H	31.350	42.293	25.199
C	33.338	41.722	25.620
N	33.366	40.995	26.796
H	32.560	41.057	27.591
C	34.354	40.110	26.722
H	34.602	39.363	27.458
N	34.975	40.233	25.545
C	34.351	41.238	24.835
H	34.661	41.513	23.840
C	35.586	36.539	22.895
H	34.742	36.992	23.400
C	35.486	35.257	22.382
H	34.571	34.684	22.474
C	36.596	34.684	21.758
N	36.515	33.317	21.278
O	35.462	32.712	21.411
O	37.515	32.824	20.763
C	37.792	35.391	21.623
H	38.645	34.916	21.154

C	37.862	36.086	22.199	C	37.878	36.679	22.117
H	38.799	36.622	22.187	H	38.795	37.247	22.034
C	36.745	36.673	22.805	C	36.789	37.281	22.797
O	36.743	37.959	23.246	O	36.898	38.478	23.329
C	37.805	38.446	24.051	C	38.235	38.614	24.595
O	38.810	37.749	24.283	O	39.142	37.799	24.397
N	37.885	39.840	23.732	N	38.486	39.994	24.506
H	37.763	39.950	22.729	H	37.621	40.515	24.450
C	39.100	40.498	24.201	C	39.424	40.360	23.452
H	38.986	40.741	25.264	H	40.445	40.284	23.839
H	39.959	39.825	24.118	H	39.335	39.650	22.621
C	39.402	41.748	23.415	C	39.210	41.756	22.923
C	40.729	42.054	23.111	C	40.304	42.529	22.525
H	41.516	41.378	23.432	H	41.309	42.142	22.673
C	41.050	43.194	22.383	C	40.121	43.773	21.923
H	42.092	43.422	22.180	H	40.979	44.353	21.603
C	40.039	44.037	21.921	C	38.837	44.281	21.739
H	40.285	44.919	21.339	H	38.690	45.248	21.268
C	38.712	43.741	22.224	C	37.741	43.520	22.137
H	37.919	44.385	21.862	H	36.737	43.868	21.939
C	38.395	42.608	22.972	C	37.928	42.267	22.714
H	37.357	42.376	23.195	H	37.056	41.657	22.941
H	32.245	43.496	26.026	H	32.204	43.504	26.001
H	28.285	41.847	27.622	H	28.359	41.970	27.608
H	37.513	38.117	27.455	H	37.644	37.993	27.633

Table S17. Cartesian coordinates (in Å) of QM atoms for the transition states corresponding to the hydrolysis steps of the esterase pathway of system pNC-RCL$_A$, optimized at M06-2X(6-31+G(d,p))/MM level of theory.

TS3 (νi = 444.7i cm^{-1})				TS4 (νi = 807.4i cm^{-1})			
Atom	X	Y	Z	Atom	X	Y	Z
C	38.233	38.664	26.590	C	37.882	38.711	26.639
H	39.268	38.963	26.781	H	38.919	38.521	26.338
H	37.603	39.560	26.708	H	37.884	39.719	27.065
O	38.121	38.131	25.291	O	37.061	38.709	25.480
H	36.009	39.907	24.628	H	36.122	39.532	25.537
C	29.225	41.703	28.301	C	29.003	41.899	28.295
H	28.964	41.197	29.235	H	28.685	41.391	29.210
H	29.451	42.745	28.548	H	29.257	42.927	28.567
C	30.513	41.063	27.743	C	30.284	41.222	27.778
O	30.516	40.546	26.608	O	30.343	40.802	26.604
O	31.572	41.131	28.465	O	31.285	41.157	28.578
C	32.223	42.635	25.273	C	32.323	42.739	25.389
H	32.360	43.081	24.284	H	32.575	43.183	24.421
H	31.285	42.071	25.275	H	31.370	42.209	25.289
C	33.347	41.668	25.473	C	33.384	41.709	25.715
N	33.505	40.858	26.574	N	33.339	40.924	26.846

H	32.808	40.832	27.377		H	32.551	40.933	27.559
C	34.592	40.098	26.395		C	34.390	40.097	26.830
H	34.929	39.335	27.080		H	34.588	39.337	27.569
N	35.153	40.392	25.223		N	35.132	40.296	25.747
C	34.387	41.370	24.635		C	34.511	41.305	25.035
H	34.630	41.772	23.663		H	34.904	41.655	24.092
C	38.562	38.879	24.180		C	37.988	38.981	24.066
O	38.982	38.204	23.215		O	38.695	37.957	23.847
N	39.300	40.033	24.560		N	38.653	40.193	24.418
H	38.883	40.622	25.269		H	37.988	40.923	24.647
C	40.071	40.729	23.552		C	39.706	40.655	23.518
H	41.042	41.015	23.976		H	40.653	40.750	24.066
H	40.252	40.001	22.760		H	39.848	39.893	22.746
C	39.458	41.957	22.907		C	39.425	41.981	22.836
C	40.264	42.732	22.066		C	40.491	42.777	22.403
H	41.314	42.474	21.957		H	41.513	42.470	22.616
C	39.743	43.824	21.379		C	40.260	43.961	21.704
H	40.386	44.383	20.704		H	41.095	44.564	21.364
C	38.403	44.176	21.543		C	38.957	44.390	21.460
H	37.969	45.002	20.987		H	38.776	45.314	20.920
C	37.599	43.425	22.395		C	37.891	43.612	21.901
H	36.548	43.671	22.503		H	36.874	43.910	21.676
C	38.123	42.325	23.075		C	38.123	42.412	22.571
H	37.484	41.721	23.712		H	37.282	41.779	22.843
O	36.968	39.429	23.776		O	36.901	39.227	23.221
H	36.989	39.634	22.833		H	36.539	38.359	22.980
H	32.133	43.371	25.944		H	32.180	43.474	26.053
H	28.447	41.670	27.674		H	28.251	41.907	27.635
H	37.937	38.003	27.280		H	37.600	38.065	27.348

Table S18. Cartesian coordinates (in Å) of QM atoms for the transition states corresponding to the decarboxylation steps of the esterase pathway of system pNC-RCL$_A$, optimized at M06-2X(6-31+G(d,p))/MM level of theory.

TS5 (vi = 347.7 cm^{-1})					TS6 (vi = 1045.0i cm^{-1})			
Atom	X	Y	Z		Atom	X	Y	Z
C	37.391	37.990	26.588		C	37.387	38.245	26.704
H	38.398	38.258	26.249		H	38.380	38.376	26.256
H	36.853	38.934	26.747		H	37.046	39.250	26.989
O	36.722	37.150	25.665		O	36.468	37.617	25.828
H	36.773	37.601	24.806		H	36.468	38.038	24.954
C	28.992	41.305	28.641		C	29.245	41.633	28.254
H	28.708	40.631	29.456		H	28.995	41.103	29.177
H	29.175	42.290	29.076		H	29.472	42.668	28.524
C	30.327	40.806	28.040		C	30.530	41.005	27.664
O	30.354	40.321	26.885		O	30.515	40.518	26.512
O	31.379	40.936	28.743		O	31.591	41.053	28.372
C	31.900	42.567	25.576		C	32.221	42.728	25.264

H	31.979	43.080	24.613
H	30.974	41.984	25.570
C	33.048	41.598	25.631
N	33.256	40.678	26.632
H	32.635	40.581	27.457
C	34.311	39.915	26.304
H	34.653	39.075	26.893
N	34.814	40.300	25.136
C	34.034	41.352	24.708
H	34.218	41.852	23.769
C	37.763	39.535	23.768
O	38.737	38.821	24.005
N	37.875	40.871	23.508
H	37.137	41.263	22.938
C	39.195	41.480	23.421
H	39.547	41.710	24.436
H	39.892	40.755	22.992
C	39.188	42.725	22.567
C	40.331	43.033	21.828
H	41.195	42.381	21.921
C	40.377	44.157	21.009
H	41.270	44.378	20.436
C	39.278	45.009	20.936
H	39.310	45.894	20.310
C	38.145	44.728	21.692
H	37.292	45.395	21.634
C	38.096	43.593	22.504
H	37.203	43.392	23.088
O	36.516	39.076	23.770
H	35.748	39.692	24.404
H	31.809	43.259	26.293
H	28.244	41.360	27.980
H	37.447	37.511	27.465

H	32.306	43.233	24.298
H	31.335	42.086	25.244
C	33.436	41.840	25.386
N	33.579	40.860	26.338
H	32.871	40.672	27.078
C	34.749	40.231	26.145
H	35.053	39.367	26.718
N	35.404	40.766	25.111
C	34.579	41.767	24.628
H	34.844	42.354	23.760
C	37.923	38.914	23.562
O	39.012	38.506	23.960
N	37.642	40.413	23.733
H	37.240	40.679	22.830
C	38.844	41.245	24.011
H	38.763	41.658	25.016
H	39.702	40.572	24.020
C	39.044	42.334	22.983
C	40.286	42.503	22.365
H	41.112	41.852	22.632
C	40.463	43.490	21.397
H	41.433	43.638	20.936
C	39.396	44.297	21.016
H	39.514	44.995	20.190
C	38.161	44.154	21.643
H	37.325	44.769	21.335
C	37.993	43.186	22.630
H	37.027	43.081	23.117
O	36.921	38.354	23.088
H	36.609	40.532	24.498
H	32.069	43.422	25.968
H	28.459	41.615	27.636
H	37.441	37.694	27.537

TS7 (νi = 120.5i cm^{-1})			
Atom	X	Y	Z
C	37.497	38.446	26.669
H	38.542	38.603	26.366
H	37.115	39.427	26.968
O	36.736	37.948	25.571
H	36.168	38.678	25.259
C	29.089	41.542	28.398
H	28.806	40.969	29.285
H	29.290	42.568	28.717
C	30.399	40.964	27.836
O	30.428	40.488	26.684
O	31.434	41.039	28.582

C	32.163	42.708	25.477
H	32.308	43.195	24.505
H	31.250	42.105	25.421
C	33.324	41.765	25.676
N	33.412	40.887	26.725
H	32.689	40.799	27.479
C	34.526	40.149	26.556
H	34.796	39.342	27.224
N	35.190	40.492	25.459
C	34.436	41.508	24.903
H	34.720	41.986	23.974
C	38.427	38.208	23.353
O	39.358	38.082	24.066
N	38.057	40.338	23.531
H	37.642	40.525	22.621
C	39.277	41.136	23.727
H	39.333	41.440	24.777
H	40.145	40.493	23.544
C	39.364	42.331	22.796
C	40.558	42.613	22.127
H	41.436	42.006	22.327
C	40.619	43.641	21.189
H	41.543	43.845	20.659
C	39.488	44.402	20.909
H	39.511	45.128	20.101
C	38.308	44.167	21.611
H	37.422	44.749	21.389
C	38.249	43.139	22.549
H	37.313	42.942	23.067
O	37.589	37.922	22.577
H	37.337	40.500	24.237
H	32.009	43.414	26.168
H	28.335	41.538	27.741
H	37.464	37.848	27.471

Table S19. Cartesian coordinates (in Å) of QM atoms for the transition state corresponding to the first acylation step of system pNC-RCL$_B$, optimized at M06-2X(6-31+G(d,p))/MM level of theory.

TS1 (νi = 772.1i cm^{-1})			
Atom	X	Y	Z
C	42.17	43.38	30.92
H	41.63	44.21	31.37
H	41.63	43.08	30.01
O	43.49	43.79	30.57
H	43.60	43.41	29.41
C	43.10	37.46	23.76
H	43.06	36.51	24.29

H	42.12	37.65	23.32
C	43.40	38.61	24.76
O	42.79	38.62	25.87
O	44.20	39.51	24.44
C	43.54	42.40	24.55
H	43.76	43.41	24.19
H	44.33	41.73	24.19
C	43.54	42.39	26.05
N	43.41	41.24	26.80
H	43.29	40.27	26.43
C	43.43	41.56	28.10
H	43.38	40.85	28.91
N	43.57	42.87	28.25
C	43.63	43.41	26.98
H	43.71	44.47	26.81
C	40.98	46.59	28.26
H	40.73	45.59	27.92
C	40.24	47.69	27.86
H	39.37	47.59	27.23
C	40.65	48.95	28.30
N	39.89	50.11	27.89
O	40.42	51.21	27.99
O	38.75	49.95	27.46
C	41.75	49.13	29.14
H	42.03	50.12	29.47
C	42.46	48.02	29.56
H	43.31	48.13	30.24
C	42.08	46.74	29.11
O	42.73	45.61	29.41
C	43.54	45.52	30.64
O	42.99	45.87	31.70
N	44.84	45.88	30.37
H	45.25	45.57	29.50
C	45.69	46.50	31.38
H	45.99	47.49	31.02
H	45.05	46.66	32.25
C	46.94	45.73	31.77
C	47.23	44.45	31.30
H	46.50	43.93	30.68
C	48.44	43.84	31.63
H	48.66	42.85	31.24
C	49.36	44.49	32.44
H	50.32	44.04	32.67
C	49.05	45.75	32.96
H	49.76	46.26	33.61
C	47.85	46.37	32.62

H	47.63	47.37	32.99
H	42.69	42.07	24.15
H	43.77	37.43	23.01
H	42.16	42.60	31.55

Table S20. Cartesian coordinates (in Å) of QM atoms for the transition state corresponding to the second acylation step of the amidase pathway of system pNC-RCL$_B$, optimized at M06-2X(6-31+G(d,p))/MM level of theory.

TS2^N (vi = 892.6i cm^{-1})			
Atom	X	Y	Z
C	38.086	38.472	26.575
H	39.145	38.245	26.410
H	38.007	39.556	26.753
O	37.314	38.116	25.440
H	36.571	40.429	24.736
C	29.219	42.024	28.233
H	28.969	41.575	29.198
H	29.472	43.071	28.427
C	30.488	41.325	27.679
O	30.497	40.912	26.497
O	31.514	41.244	28.436
C	32.407	42.893	25.339
H	32.544	43.417	24.389
H	31.501	42.283	25.274
C	33.585	41.971	25.494
N	33.713	41.040	26.501
H	32.982	40.898	27.228
C	34.863	40.365	26.323
H	35.193	39.543	26.941
N	35.501	40.822	25.247
C	34.707	41.824	24.719
H	34.973	42.357	23.819
C	35.725	36.132	22.704
H	34.857	36.646	23.101
C	35.624	34.863	22.158
H	34.678	34.338	22.116
C	36.772	34.255	21.661
N	36.658	32.915	21.088
O	35.600	32.327	21.211
O	37.634	32.455	20.509
C	38.017	34.877	21.710

TS3^N (vi = 88.9i cm^{-1})			
Atom	X	Y	Z
C	38.078	38.526	26.474
H	39.160	38.404	26.391
H	37.838	39.592	26.542
O	37.424	38.019	25.304
H	37.326	40.618	24.524
C	29.079	41.900	28.328
H	28.782	41.406	29.258
H	29.317	42.938	28.583
C	30.377	41.239	27.815
O	30.431	40.819	26.638
O	31.375	41.186	28.609
C	32.375	42.838	25.497
H	32.533	43.329	24.530
H	31.455	42.247	25.443
C	33.531	41.915	25.732
N	33.588	41.015	26.763
H	32.821	40.873	27.444
C	34.778	40.372	26.678
H	35.058	39.581	27.360
N	35.503	40.806	25.661
C	34.724	41.773	25.064
H	35.052	42.313	24.186
C	35.864	36.065	22.575
H	35.051	36.677	22.948
C	35.650	34.789	22.075
H	34.660	34.352	22.045
C	36.743	34.058	21.623
N	36.510	32.702	21.110
O	35.395	32.238	21.231
O	37.449	32.120	20.591
C	38.040	34.552	21.665

H	38.896	34.370	21.329		H	38.870	33.947	21.320
C	38.114	36.149	22.259		C	38.254	35.828	22.173
H	39.064	36.655	22.334		H	39.253	36.231	22.257
C	36.966	36.781	22.759		C	37.163	36.569	22.626
O	36.956	38.054	23.221		O	37.301	37.852	23.111
C	37.954	38.488	24.203		C	38.153	38.021	24.179
O	39.141	38.086	24.070		O	39.346	37.807	24.130
N	37.728	40.036	24.036		N	38.021	40.256	23.863
H	37.473	40.114	23.049		H	37.610	40.275	22.934
C	38.990	40.793	24.214		C	39.316	40.924	23.879
H	39.080	41.089	25.264		H	39.586	41.117	24.925
H	39.811	40.104	24.002		H	40.058	40.218	23.484
C	39.143	42.003	23.317		C	39.425	42.210	23.074
C	40.426	42.360	22.883		C	40.672	42.609	22.580
H	41.274	41.742	23.163		H	41.548	41.995	22.778
C	40.628	43.479	22.081		C	40.802	43.768	21.821
H	41.632	43.754	21.776		H	41.776	44.066	21.447
C	39.544	44.256	21.673		C	39.686	44.550	21.532
H	39.694	45.114	21.024		H	39.780	45.439	20.916
C	38.265	43.907	22.092		C	38.444	44.165	22.025
H	37.414	44.483	21.751		H	37.566	44.747	21.776
C	38.068	42.797	22.914		C	38.315	43.007	22.793
H	37.060	42.540	23.226		H	37.333	42.713	23.152
H	32.253	43.570	26.059		H	32.238	43.537	26.198
H	28.423	41.984	27.630		H	28.318	41.886	27.679
H	37.746	37.998	27.387		H	37.728	38.060	27.287

5. References

(1) Eisenberg, D.; Schwarz, E.; Komaromy, M.; Wall, R. Analysis of membrane and surface protein sequences with the hydrophobic moment plot. *Journal of Molecular Biology* **1984**, *179* (1), 125–142. DOI: 10.1016/0022-2836(84)90309-7.

(2) Field, M. J., Bash, P. A., Karplus, M. A combined quantum mechanical and molecular mechanical potential for molecular dynamics simulations. *J. Comput. Chem.* **1990**, *11* (6), 700–733. DOI: 10.1002/jcc.540110605.
